# Genome-wide identification of microsatellites in white clover (*Trifolium repens *L.) using FIASCO and phpSSRMiner

**DOI:** 10.1186/1746-4811-4-19

**Published:** 2008-07-16

**Authors:** Yan Zhang, Ji He, Patrick X Zhao, Joseph H Bouton, Maria J Monteros

**Affiliations:** 1 Forage Improvement Division, The Samuel Roberts Noble Foundation, 2510 Sam Noble Parkway, Ardmore, OK, 73402, USA; 2 Plant Biology Division, The Samuel Roberts Noble Foundation, 2510 Sam Noble Parkway, Ardmore, OK, 73402, USA

## Abstract

**Background:**

Allotetraploid white clover (*Trifolium repens *L.) is an important forage legume widely cultivated in most temperate regions. Only a small number of microsatellite markers are publicly available and can be utilized in white clover breeding programs. The objectives of this study were to develop an integrated approach for microsatellite development and to evaluate the approach for the development of new SSR markers for white clover.

**Results:**

Genomic libraries containing simple sequence repeat (SSR) sequences were constructed using a modified Fast Isolation by AFLP of Sequences COntaining repeats (FIASCO) procedure and phpSSRMiner was used to develop the microsatellite markers. SSR motifs were isolated using two biotin-labeled probes, (CA)_17 _and (ATG)_12_. The sequences of 6,816 clones were assembled into 1,698 contigs, 32% of which represented novel sequences based on BLASTN searches. Approximately 32%, 28%, and 16% of these SSRs contained hexa-, tri-, and di-nucleotide repeats, respectively. The most frequent motifs were the CA and ATG complementary repeats and the associated compound sequences. Primer pairs were designed for 859 SSR loci based on sequences from these genomic libraries and from GenBank white clover nucleotide sequences. A total of 191 SSR primers developed from the two libraries were tested for polymorphism in individual clones from the parental genotypes GA43 ('Durana'), 'SRVR' and six F_1 _progeny from a mapping population. Ninety two percent produced amplicons and 66% of these were polymorphic.

**Conclusion:**

The combined approach of identifying SSR-enriched fragments by FIASCO coupled with the primer design and *in silico *amplification using phpSSRMiner represents an efficient and low cost pipeline for the large-scale development of microsatellite markers in plants.

The approach described here could be readily adapted and utilized in other non-related species with none or limited genomic resources.

## Background

Microsatellite or SSR markers are PCR-based markers widely used in mapping and QTL detection studies in plants [1]. A number of methods have been used to identify microsatellite markers including a Fast Isolation by AFLP of Sequences COntaining repeats (FIASCO) approach [2]. FIASCO is a fast and effective technique to develop SSR markers compared to methods previously used [3,4]. Some of the advantages of FIASCO are smaller genomic DNA (250 ng) requirements and the use of only one restriction enzyme, improved SSR selection and library enrichment procedures, significantly simplified cloning, transformation, and colony selection.

FIASCO has been used for SSR development in many different organisms such as birds (*Passera lagia*), fish (*Sparus aurata *and *Lophius americanus*), crustaceans (*Meganyctiphanes norvegica*), and red coral (*Corallium rubrum*) [2]. Due to the relative ease of using FIASCO, this method was also used to develop SSR markers in a number of less studied species including fungal pathogens and insects [5-7]. Recently, FIASCO has also been used in plant species. For example, [8] described the characterization of 13 microsatellite loci using FIASCO in the genome of an important Chinese herbal medicine, *Gastrodia elata *Blume. Eleven polymorphic microsatellite markers were identified using the same method in the endangered shrub *Ammopiptanthus mongolicus *[9]. FIASCO was also used to develop 24 SSR markers from the sacred lotus (*Nelumbo nucifera *Gaertn.), an important economic and ornamental aquatic plant in China [10]. These studies indicate the potential application of FIASCO in SSR development in plant species even though this technique has not been utilized to identify a large number of genome-wide SSRs.

White clover (*Trifolium repens *L.) is an allopolyploid (2n = 4x = 32) outcrossing species [11] extensively used as a cool season forage legume worldwide. The breeding objectives of white clover are mainly focused on improving the persistence, dry matter yield, stolon density, disease resistance, digestibility, seed yield, and competitive ability [12]. Many improvements have been achieved in white clover using a conventional breeding approach [13]. The use of molecular markers can significantly accelerate breeding and selection efficiency and have been utilized to target complex traits in many species including white clover [14,15]. Although white clover linkage maps are available [16-18] and a few quantitative trait loci (QTL) have been identified using SSRs [14,15], only a small number of these markers are publicly available and can be utilized in white clover breeding programs outside the groups that developed them.

We report the large scale development and characterization of genome-wide SSR markers in white clover from two genomic libraries enriched for (CA)_17 _and (ATG)_12 _repeats using the FIASCO method, and from white clover sequences available in GenBank using the user-friendly bioinformatics software phpSSRMiner. Evaluation of polymorphism for 191 SSR markers was conducted using genomic DNA from two white clover parent clones and six F_1 _plants resulting from that cross. The markers developed will greatly enhance the resolution of white clover genome mapping studies and facilitate future marker-assisted selection applications.

## Methods

### Digestion-ligation and PCR amplification

The GA02-15 clone from the SRVR germplasm [19], one of the parental plants of a mapping population [18], was used to construct the SSR-enriched libraries. An initial PCR reaction in a 25 μl reaction contained 250 ng of genomic DNA, 25 μM of NaCl, 1 unit of T4 ligase (Promega, Madison, WI, USA), 1× T4 DNA ligase buffer (Promega, Madison, WI, USA), 25 μg/ml BSA (New England Biolabs, Boston, MA, USA), 2.5 units of *Mse*I, and 2 pmol of adapter. To make the adapters, 1 μM of each of two oligonucleotides (5' GACGATGAGTCCTGAG 3' and 5' TACTCAGGACTCAT 3') were combined and heated at 95°C for 5 min in a MJ Research thermal cycler. These were allowed to cool at room temperature for 15 min to allow the two sequences to anneal together and form the adapters. An initial digestion-ligation of genomic DNA of the SRVR clone with *Mse*I (New England Biolabs, Boston, MA, USA) and the adapters was incubated at 37°C for 3 hours, and then directly amplified using the adapter-specific primers *Mse*I-N (5'-GATGAGTCCTGAGTAAN-3'), which contained equal amounts of all four possible selective bases (N = A, T, C, or G). This permits the amplification of all fragments flanked by *Mse*I sites. PCR reactions were performed in a total volume of 20 μl: 1.5 mM MgCl_2 _(Applied Biosystems, Foster City, CA, USA), 30 ng of each of the four *Mse*I-N primers, 200 μM each of dNTPs (GE Healthcare, Piscataway, NJ, USA), 1× *Taq *DNA polymerase buffer (Promega, Madison, WI, USA), 0.4 units of *Taq *DNA polymerase (Promega, Madison, WI, USA), and 5 μL of a 1:10 dilution of digested-ligated DNA. PCR conditions used were: 94°C 30 s, 53°C 1 min, 72°C 1 min, and 18, 20 and 23 cycles were tested to determine the optimal number of amplification cycles.

### Hybridization with biotin probes

A total of 500 ng of amplified DNA using 20 PCR cycles were added to 200 pmol of HPLC-purified biotinylated oligonucleotide (AG)_17 _or (ATG)_12 _and combined in a total volume of 100 μL of SSC 4.2×, SDS 0.07%. The DNA mixture was denatured at 95°C for 3 min and allowed to anneal at room temperature for 15 min.

### Bead preparation and DNA capture

One mg of streptavidin-coated magnetic particle beads (Roche Applied Science, Indianapolis, IN, USA) was washed in TEN_100 _buffer (10 mM Tris-HCl, 1 mM EDTA, 100 mM NaCl, pH 7.5) and re-suspended in 40 μl of the same buffer. Ten μl (corresponding to approximately 1 μg of DNA) of tall fescue PCR product using primers from the actin gene, were combined with the beads to minimize nonspecific binding of white clover genomic DNA. DNA-probe hybrid molecules were diluted with 300 μL of TEN_100_, and combined with the prepared beads. A 450 μl DNA-probe-bead solution was incubated for 30 min at room temperature with constant gentle agitation. The hybridization buffer was separated from the beads-probe-DNA complex using a Dynabeads magnetic separation stand (Invitrogen, Carlsbad, CA, USA) and discarded. Washing of DNA-probe and DNA collection were performed as previously described [2].

### DNA precipitation and cloning

Recovered DNA fragments from three separate wash steps were precipitated with one volume of isopropanol and sodium acetate (0.15 M final concentration), and re-suspended in 50 μl of water. Two μl of the recovered fraction were amplified with 30 cycles of PCR using the *Mse*I-N primer using the same conditions described above. Four separate PCR reactions were conducted, each one with one primer (*Mse*I-A, or T, or G, or C) removed from the primer mixture to select the DNA populations with the least amount of duplicated amplification. The amplified fragments with the selected primers produced a smear when visualized in an ethidium-bromide-stained agarose gel. To check for complete removal of nonspecifically bound DNA, 2 μl of the last fraction recovered was amplified with the same primer sets and did not yield any product. The resulting PCR products were cloned into TOPO TA cloning vector and transformed with One Shot TOP10F' chemically competent cells (Invitrogen, Carlsbad, CA, USA) following the manufacturer's instructions. Colony PCR was conducted on 16 clones per library using universal M13 primers to examine the inserts.

### Sequencing and data assembly

Sequencing and data analysis were conducted at the Samuel Roberts Noble Foundation. Briefly, white colonies were cultured in 96-well culture blocks containing 1.5 ml TB and a salt supplement (TE-RNase A+T1, SDS/NaOH, 3 M NaOAc, pH 4.8) together with 100 μg/ml ampicillin for 22 hours at 37°C while shaking at 350 rpm. Double-stranded DNA template was isolated as described . All sequencing reactions were performed using the flanking M13 reverse primer site and analyzed in an ABI 3730 capillary sequencer using the BigDye^® ^Terminator Sequencing Kit (Applied Biosystems, Foster City, CA, USA). Base calling of the ABI sequencer trace files was done with the vendor program which was essentially based on Phred . Each sequence was then processed using an in-house semi-automated pipeline for overall base quality screening and removal of any contaminating vector, mitochondrial, ribosomal and *E. coli *sequences. High quality sequences that passed this screen and having a minimum length of 100 bp were assembled into contigs and used for SSR detection. All assembled contigs were also examined for homology to a variety of public databases through batch BLASTN [20] searches.

### SSR mining and primer design

The high quality sequence contigs were analyzed using the phpSSRMiner web server , developed at The Samuel Roberts Noble Foundation. PhpSSRMiner provides a user-friendly interface which integrates its back-end pipeline to streamline the process of perfect SSR identification by SSRIT [21], imperfect SSR identification by Sputnik  developed by [22], PCR primer design using Primer3 [23], and *in silico *amplification of the designed primers via isPCR [24]. SSR identification, PCR primer design and *in silico *amplification of the designed primers were performed and hosted by phpSSRMiner. The sequence information, both from the contig sequences and GenBank, feeds directly into the software and the output is a tab-delimited text file with separate columns for the positions and sequences of the forward and reverse primers, type of SSR repeat (perfect vs. imperfect), SSR motif, and length.

### Examination of SSR polymorphism

Forward and reverse primers were synthesized by Qiagen/Operon Biotechnologies (Los Angeles, CA, USA) with an additional 18 nucleotides from the M13 universal primer appended to the 5' end of the forward primer [25]. PCR amplification and genotyping in the GA02-15 genotype from SRVR and the GA02-56 genotype from 'Durana' [26], as well as in six progeny from the resulting F_1 _mapping population, were performed following standard procedures previously described [18]. SSR fragments amplified were analyzed on the ABI PRISM 3730 Genetic Analyzer (Applied Biosystems, Foster City, CA, USA) and visualized and scored using GeneMapper 3.7 software (Applied Biosystems, Foster City, CA, USA).

## Results and Discussion

### Construction of the SSR-enriched libraries

**Figure 1:**
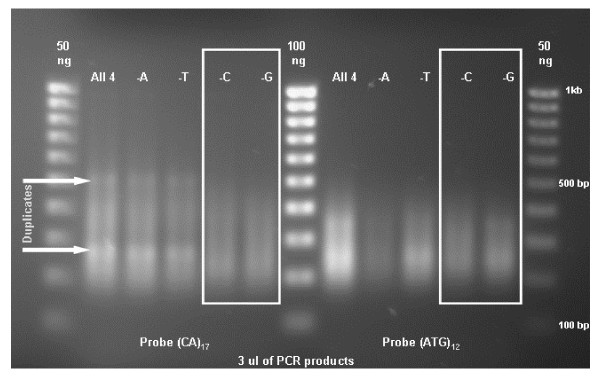
PCR products using selected primers of precipitated DNA from SSR-enriched libraries. A 1 kb molecular size standard was loaded on each side and in the middle of the gel. A total of 3 μl of PCR products were loaded in each lane to evaluate the primer combinations (all four primers vs. three primer combinations of *MseI *-A, -T, -C, or -G) showing the least duplication (bands) of PCR products. The primer combinations without *MseI *-C and -G were selected to generate the SSR-enriched libraries.

In most plant species the AT/TA and GA/CT are the most common motifs [27]. However, [28] found that CA and ATG were the most frequent repeat motifs in white clover and therefore, (CA)_17 _and (ATG)_12 _were used as the di- and tri-nucleotide repeat probes for constructing the libraries. To remove duplicate PCR products generated during amplification, the best combination of three primers (minus one of four *Mse*I-A, -T, -C, or -G) was tested to amplify SSR-containing fragments in the last step of the DNA precipitation procedure. To avoid sequencing duplicated DNA fragments, only the PCR products showing smears and not distinguishable bands were used for cloning (Figure 1). Primers without *Mse*I-G or *Mse*I-C were used to produce the PCR products of the (CA)_17_, and (ATG)_12 _probe libraries, respectively (Figure 1). In a study with wheat, the mixture of primers minus *Mse*I-A produced the most specific amplification (Mahmoud Zeid, personal communication) suggesting that the optimal primer combination might be species-specific. After removing the priming sites, the size range of the inserts for the (CA)_17 _library was 200–300 bp and 200–350 for (ATG)_12_. No double inserts were detected (data not shown).

### Genomic sequences of SSR-enriched libraries

Over 6,800 clones were generated from the two SSR-enriched libraries and sequenced. The (CA)_17 _library contained 2,208 clones and the (ATC)_12 _library contained 4,608 clones (Table 1). Approximately 56% and 66% of these clones generated successful sequencing reactions, with an average length of 238 bp and 263 bp for the two libraries, respectively. After assembling the sequences from the two libraries, 826 and 876 contigs were found in each library representing more than 61% and 43% good quality, unique sequences, indicating that the strategy of using selected primer combinations in PCR reduced duplication. A total of 1,698 contigs were found after assembling all sequences from both libraries, slightly fewer than the total number of contigs from the two libraries (Table 1). This indicates the uniqueness of the sequences selected with different probes using the FIASCO procedure, with very little overlap existing between the (CA)_17 _and (ATG)_12 _library sequences. A previous study used the Edwards method [3] to construct SSR-enriched libraries with six different restriction enzymes prior to enrichment of the libraries and generated 1,123 white clover clones with readable DNA sequences [28]. Our study generated a total of 2,689 white clover genomic DNA sequences which have been submitted to GenBank . These include 1,224 sequences from the (CA)_17 _library and 1,465 from (ATG)_12 _library. The accession numbers [GenBank: EF690813-EF691565, EU416331 – EU416801] and [GenBank: EF691566-EF692395, EU416802 – EU417436] were assigned to the sequences from the (CA)_17_, and (ATG)_12 _libraries, respectively.

**Table 1:** Description of genomic sequences obtained from both SSR-enriched libraries

**Library**	**No. of ** **clones**	**No. of** **total** ** sequences**	**No. of clean ** **sequences**	**Avg. clean** **sequence** ** length (bp)**	**No. & (percent) of ** **contigs from clean** ** sequences**	**Avg. contig sequence** **length (bp)**
(CA)_17_	2,208	2,400	1,351	238	826 (61%)	242
(ATG)_12_	4,608	3,072	2,021	263	876 (43%)	265
Combination of two libraries	6,816	5,472	3,372	253	1,698 (50%)	254

### SSR-sequence distribution

The repeat sequences identified using the phpSSRMiner software include both perfect and imperfect SSRs with a minimal size of 18 bp. A similar number of sequences containing SSRs were found in the two libraries, although these are based on different numbers of contigs (Table 2). The percentage of sequences with SSRs was higher in the (ATG)_12 _than the (CA)_17_library. Up to three repeat motifs were present in each contig and 260 and 318 SSRs were identified for the (ATG)_12 _and the (CA)_17 _library, respectively. The most frequent motif in the (CA)_17 _library was TG, followed by CA, AC, and GT (Table 2). The most common and distinguishable SSR motifs identified in the (ATG)_12 _library were GAT, TGA, CAT, and ATC. As expected, the most frequent repeats were di-nucleotides in the (CA)_17 _library and tri-nucleotides in the (ATG)_12 _library. Only 21 tri-nucleotide repeats were identified in the (CA)_17 _library and only four di-nucleotide repeats were found in the (ATG)_12 _library, indicating the high selection efficiency of FIASCO for either di- or tri-nucleotide repeats. These results suggest that the probing procedure was able to target the specific motif and their complementary strings, producing SSR-enriched libraries with the expected repeat motifs. The length of the repeat motifs ranged from 2–10 bp, with 2–34 copies of repeats. Both the di- and the tri-nucleotides were the most abundant type of repeat and represented approximately 45% of the total number of SSRs identified. Hexa-nucleotide repeats identified in both libraries were the second most frequent type of repeat detected. A total of 575 SSRs were detected in the assembled sequences. The top motifs GAT, TGA, and TG, CA in the (ATG)_12_, and (CA)_17 _libraries remain the same due to the unique sequences between the two libraries (Table 2). The motifs containing hexa-, tri-, and di-nucleotide repeats were the most common ones, accounting for 76% of all SSR motifs identified (Table 2).

**Table 2:** Summary of SSR distribution from white clover SSR-enriched libraries

**Library**	**No. of ** **sequences**	**No. of** **sequences** ** with SSRs^a^**	**Percentage of ** **sequence with ** **SSRs**	**No. of ** **SSRs**	**Most frequent core** **motifs**	**Motif ** **length ** **(bp)**	**No. of ** **repeat**	**Most frequent ** **repeats- No. &** **(percentage)**
(CA)17	826	191	23%	260	TG, CA, AC, GT	2–10	2–31	Di- 90 (35%)
								Hexa- 68 (26%)
(ATG)12	876	230	26%	318	GAT, TGA, CAT, ATC	2–10	2–34	Tri- 138 (43%)
								Hexa- 118 (37%)
Assembled	1,698	419	25%	575	GAT, TGA, TG, CA	2–10	2–34	Hexa- 186 (32%)
								Tri- 159 (28%)
								Di- 92 (16%)

^a ^Number of both perfect and imperfect SSRs, with a minimal length of 18 bp.

### Sequence homology search in other databases

BLASTN searches of the 1,698 contig sequences against 1,983 white clover nucleotide sequences available in GenBank using an e-value threshold of 1e^-20 ^indicated that only 123 sequences generated in this study had homology to GenBank white clover nucleotide sequences (Table 3). These sequences accounted for only 7.2% of the 1,698 sequences obtained from the libraries, indicating that more than 92% of the identified white clover sequences were unique. BLASTN searches against the NCBI non-redundant nucleotide library (NCBI NT) using a less stringent threshold of 1e^-10 ^indicate that approximately 68% of our white clover sequences have matches and the remaining one third of the contigs represent novel white clover sequences. BLASTN and TBLASTX searches against the *Medicago truncatula *databases (BAC and EST) resulted in matches for only 17% and 9% of the identified sequences in the IMGAG *Medicago *Pseudo Genome  and *M. truncatula *Gene Index from TIGR (MtGI, ), respectively. The low percent of sequence similarity between *M. truncatula *and these white clover sequences, as well as results from a previous study indicating the relatively low amplification rate of white clover genome sequences using *M. truncatula *EST-SSRs [18] further enhances the importance of these primer sequences.

**Table 3:** Number and percentage of white clover genomic sequences with homology to white clover sequences and public databases with *Medicago truncatula *sequences

**White clover ** **sequences NF^a^**	**White clover sequences ** **GenBank**	**NCBI NT**	***M. truncatula *pseudo** **genome**	***M. truncatula *gene index**
1,698	123 (7.2%)	1,152 (67.8%)	288 (17.0%)	156 (9.2%)

The e value thresholds for homology searches used were 1e-^20 ^for GenBank and 1e-^10 ^for the NCBI non-redundant nucleotide library (NCBI NT), *M. truncatula *pseudo genome, and *M. truncatula *gene index.

^a ^Number of contigs generated from the two SSR-enriched genomic libraries

### Generation of SSR primers from T. repens nucleotide sequences

As of Jan 22 '08, 4,672 white clover nucleotide sequences were available in GenBank. A total of 2,689 (58%) of them were generated in this study from the SSR-enriched genomic libraries. The remaining 1,983 white clover sequences were downloaded from GenBank and loaded into phpSSRMiner for microsatellite primer design. The most frequent motif repeats from GenBank sequences were CA and TG. These were also the most frequent motifs in the white clover sequence from the libraries developed in this study (Table 2). After removing the 102 GenBank sequences having high homology with the sequences obtained from the two genomic libraries, 1,881 sequences were used to design 597 primers that included 230 perfect and 367 imperfect SSR primers. The phpSSRMiner analysis also identified 262 SSR primers from the 1,698 white clover sequences generated in this study. From those, 22% (58) were perfect primers, indicating that they amplify perfect repeats of the motif, and 78% (204) were imperfect primers. Imperfect primers allow a one or two base pair insertion in the repeat motif. In total, 859 primer pairs were identified (see Additional file 1: Characteristics of 859 SSR primers developed using GenBank white clover nucleotide sequences and two genomic SSR-enriched libraries generated in this study) and their location is currently being mapped in a white clover population. A smaller number of SSR primers were generated from the two genomic libraries compared to GenBank database sequences even though a similar number of sequences were contained in each data set. The main reason for this difference was the length of the sequences. The average length of the 1,881 white clover sequences from GenBank was 813 bp compared to the 254 bp average of the contigs from the two genomic libraries. This difference in the length of the sequences affects the likelihood of identifying potential priming sites.

### SSR polymorphism

PCR amplification in the laboratory with a subset of randomly selected 191 primer pairs flanking 63 perfect and 128 imperfect SSRs designed using the phpSSRMiner software was used to verify amplification products of the expected size. DNA from the parents of a mapping population (GA43 and SRVR), as well as six F_1 _progeny from a double-pseudo testcross [18] was used to determine the polymorphisms of these SSR primers (Table 4). A total of 176 (92%) of the primers screened produced amplicons, 66% of which were polymorphic between the two parents.

**Table 4:** Summary of SSR primer screening based on laboratory PCR amplification

**Total primers tested**	**Perfect SSR primers**	**Imperfect SSR primers**	**Primers with amplicons**	**Monomorphic primers**	**Polymorphic primers**	**Polymorphic perfect SSR primers**	**Polymorphic imperfect SSR primers**
191	63	128	176 (92%^a^)	60 (31%^b^)	116 (66%^b^)	47 (75%^c^)	69 (54%^c^)

^a ^percentage of designed primers with PCR amplification products

^b ^percentage of primers with amplicons in GA43, SRVR, and six F1 progeny

^c ^percentage of polymorphic primers within the perfect and imperfect groups

**Figure 2:**
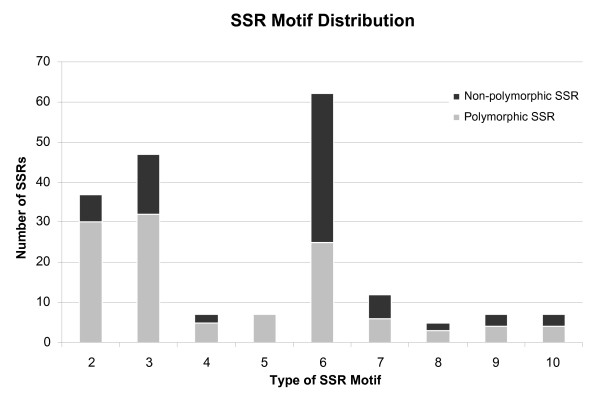
Distribution and polymorphism of 191 white clover SSRs based on number of repeat units (i.e. 2 = di-, 3 = tri, 6 = hexa- nucleotide repeats). The polymorphism was tested in GA43, SRVR, and six F_1 _progeny.

Primers were designed to amplify sequences containing both perfect and compound SSRs. For the 63 perfect and 128 imperfect SSRs, the percentage of primers amplifying polymorphic fragments was higher for the perfect (75%) vs. the imperfect SSRs (54%) (Table 4). Among the 191 primers, 33% of them were from hexa-nucleotide SSRs, followed by 25%, and 19% from tri- and di-nucleotide SSRs, respectively (Figure 2). Even though primers amplifying di-nucleotide SSRs were not the most abundant, they gave the highest percentage of polymorphism (Figure 2). Our results suggest that the FIASCO procedure can be utilized to develop molecular markers in allopolyploid genomes with homoeologs of high sequence identity, such as white clover.

## Conclusion

We report the development of white clover SSR markers using the FIASCO technique to develop SSR-enriched libraries coupled with the use of the phpSSRMiner software to design primers and verification of amplification *in silico*. The combined approach represents an efficient and low cost pipeline for the large-scale development of microsatellite markers in plants. The publicly available process described here to obtain SSR primer sequences would be useful for species that have limited genomic resources. The white clover SSRs developed will be integrated into an existing genetic linkage map [18] and current efforts include evaluation of their potential for cross-species amplification across forage legume species to use in comparative mapping studies. Increasing the number of markers in white clover linkage maps will improve the resolution of maps used for mapping QTL for traits of interest. By making these primer sequences publicly available, we hope to encourage the development of white clover consensus linkage maps that will help to overcome some of the limitations in comparing mapping results generated by research groups from around the world utilizing different and often proprietary markers. The availability of markers with transferability across studies and populations will advance the utilization of markers in diverse breeding programs with different genetic backgrounds and facilitate progress towards the common goal of developing improved white clover cultivars.

## List of abbreviations

FIASCO: Fast Isolation by AFLP of Sequences Containing repeats; SSR: simple sequence repeat; QTL: Quantitative Trait Loci.

## Competing interests

The authors declare that they have no competing interests.

## Authors' contributions

YZ experimental design, construction of genomic libraries, sequence data mining and analysis, SSR primer development and polymorphisms screening, and manuscript preparation. JH sequence data mining and analysis, SSR primer development. PXZ sequence data mining and analysis, SSR primer development. JHB providing plant materials, experimental design. MJM data analysis, interpretation, and manuscript preparation and revision. All authors read and approved the final manuscript.

## Supplementary Material

Additional file 1 Characteristics of 859 SSR primers developed using GenBank white clover nucleotide sequences and two genomic SSR-enriched libraries generated in this study. Click here for file
