# Supplementary material for: Genome-wide identification of microsatellites in white clover (Trifolium repens L.) using FIASCO and phpSSRMiner
Source: Plant Methods. 2008 Jul 16;4:19. doi: 10.1186/1746-4811-4-19 (PMC2517061; doi:10.1186/1746-4811-4-19)
Supplement: Additional file 1 — Characteristics of 859 SSR primers developed using GenBank white clover nucleotide sequences and two genomic SSR-enriched libraries generated in this study. [file 1746-4811-4-19-S1.doc]

**Additional file 1**

Characteristics of 859 SSR primers developed using GenBank white clover nucleotide sequences and two genomic SSR-enriched libraries generated in this study. The source of the primers identified is included as a prefix in the primer ID. GenBank = white clover nucleotide sequences available in GenBank; NF = sequences are from the two white clover genomic SSR-enriched libraries.

| **Primer ID** | **SSR type** | **SSR motif** | **SSR motif length** | **SSR # repeat** | **SSR length** | **Forward primer** | **Reverse primer** |
| --- | --- | --- | --- | --- | --- | --- | --- |
| GB208808 | Perfect | AT | 2 | 21 | 42 | AAAATGAATGGAAGGAAGAAA | TTTCAAAATTTCTCCAACATCTCTC |
| GB208861 | Perfect | ATTTTT | 6 | 3 | 18 | CATCGTTAATTTTCATCTTTCAA | AATGGTTCGGTTTGGTTTCTC |
| GB208896 | Perfect | TTGTGAC | 7 | 3 | 21 | TGTATTAATATCAACTATTAGGCTG | AAAAATGGATAAACAGATAAGTGA |
| GB208995 | Perfect | AATGCTTCT | 9 | 2 | 18 | TTCAGTTTCTTTCCAAACTTGTCA | GTTGGCATTTCGCTTATTGAA |
| GB209060 | Perfect | TTGGAAACT | 9 | 2 | 18 | GGTTTCAGGCATGTGATGCTA | TCTCATTTTTCTCTGCTCCCA |
| GB209108 | Perfect | TAAAAGAGT | 9 | 2 | 18 | AATCAGGAGATATAAGGTTGGTTT | TTGAACAATCTACTCATAGCAGGA |
| GB209126 | Perfect | ATATAC | 6 | 3 | 18 | AACTTTCACCATAATCAATCATCA | CACCCGGAACCTATTTTCAAT |
| GB209159 | Perfect | TCAAGAAAA | 9 | 2 | 18 | GGCAAATTCCTAGAGAAATGAA | ATCGTGCTACGATGAACGG |
| GB209175 | Perfect | AAACT | 5 | 4 | 20 | TCATGAACGTCTTTAAATGAACAA | ATTCTTTGAAGGCATGGGACT |
| GB209181 | Perfect | GTGGTGCTC | 9 | 2 | 18 | AAAATCCAAACGCTGCTTCAT | TTACCTCGTGAGAAAGGAGGG |
| GB209185 | Perfect | GGCGGT | 6 | 3 | 18 | AGAAGGTTCTGGTGGTGGTGT | GGATGGATCAAAGAAACAAAGC |
| GB209203 | Perfect | TCA | 3 | 6 | 18 | CCCAATTCCACAACTCAAACTT | TCAGGGTCATCACCTATGTCAG |
| GB209210 | Perfect | AGA | 3 | 6 | 18 | GGCCCTAGTAGAAAGCCTAGC | CATCACTCAATTCCCTCTCCA |
| GB209224 | Perfect | TTTTTGTTTT | 10 | 2 | 20 | AAAAGAGTGTAATAGGTAAGAATGTT | GGGTCAGATTCGGTATGTTCC |
| GB209236 | Perfect | AACAGTAGG | 9 | 2 | 18 | CACGTGTAGAACTTTGATAGGA | CCGGTGTACTGATTAAATATGCC |
| GB209256 | Perfect | TAGATAAAT | 9 | 2 | 18 | TCACCTGTAGTTATTGTAATCGTT | CCCACCACCGGTTATTATTTC |
| GB209257 | Perfect | TTCA | 4 | 7 | 28 | ATACATACGTGTGAAAACAACTTT | TTTGCTCGTGTAGAAGAAGAAGT |
| GB209281 | Perfect | AAAAAAATCA | 10 | 2 | 20 | CAGAGTTTTCTTTTTACAAATTGC | CACCTAGCAGCAGAAAATGGA |
| GB209287 | Perfect | CTT | 3 | 7 | 21 | CCGTTCAGAAGGAAGAAAAGAA | ATTCAACTTCCCGCTCTTCAT |
| GB209291 | Perfect | ATAATTAGAT | 10 | 2 | 20 | TGAAGGAGAGAATAAGAACAAGCA | TTGAAGTATGGGATGGAATTTTT |
| GB209297 | Perfect | AGCTTAT | 7 | 3 | 21 | AGCTTATTTTTCAAACGCTATTTCA | ATGAAAATGAAACCACGGAAA |
| GB209306 | Perfect | TGTTTTATC | 9 | 2 | 18 | GCACTTGCCTATGAAAATCCA | TTAAGTATTTGCTCGGCTCCA |
| GB209340 | Perfect | AAAAACTTA | 9 | 2 | 18 | TTGGAGGAGATTAATTTAAAGGG | CTAAATTTATCCAAAGGTCTTTTA |
| GB209406 | Perfect | TA | 2 | 20 | 40 | AGAACAGGAGTTGGACGCAAT | TTGCCATCGATTTCTGCTAAT |
| GB209412 | Perfect | TCT | 3 | 6 | 18 | GTTGTCGGTGGTCAGGTAACA | TACCAGATCCACGAGGAAGAA |
| GB209434 | Perfect | ATA | 3 | 6 | 18 | TCCTTATACAATGAACTATTGACGTT | ATATATGAAGCCCCGATACGC |
| GB209469 | Perfect | CTTTTCCTT | 9 | 2 | 18 | AAAGAGCTTTGTGCAGCTTGT | TTAATGAAACTTTCGCTCCGA |
| GB209470 | Perfect | ATCT | 4 | 5 | 20 | AAGGTTGGGCATCAGTTCAG | TGAATACAAACCTGTGTGGCA |
| GB209506 | Perfect | CAGGAGGAG | 9 | 2 | 18 | GAATCAAAATGTATTGCCGGA | CCTGTCAATTTTCATCAACCG |
| GB209517 | Perfect | AGCACATGT | 9 | 2 | 18 | CTGGCATCACATCATGTACCA | TAGCTAAAAGGGGGAAAAA |
| GB209540 | Perfect | AAAAATGAT | 9 | 2 | 18 | GGCACACTTACAATAAAGGTGAAA | AAGCACAAGCAGTGCTAGGAG |
| GB209543 | Perfect | AAT | 3 | 6 | 18 | CCCTTTACTCTTTTCAATTTTCA | CAACTTCAAATTTTCTCCCCC |
| GB209547 | Perfect | ACATAAAAA | 9 | 2 | 18 | GCATTCTTTCCTGTATTTCAAGTG | GCTTATATTTTGGGACGGAGG |
| GB209548 | Perfect | AT | 2 | 24 | 48 | TTCAGTTTTAAAGGCAATTCTCAA | ACCGTTAATCTTGATGGGTCC |
| GB209552 | Perfect | TA | 2 | 9 | 18 | ATGGGAGCGGATAATTTCCTA | ACATGTTTGTTGTTGGCTTCC |
| GB209575 | Perfect | CTA | 3 | 18 | 54 | TTTGTGGCGGTTTCTATTGAT | TGACTTCTTATATGCCCGCAC |
| GB209598 | Perfect | GT | 2 | 10 | 20 | AAGGTGATATGATCTGAAATGGAA | ACACTGACACGTCAACACCAA |
| GB209631 | Perfect | GA | 2 | 14 | 28 | ATCATGTGGAAAAGATGTAGAAA | TGGGGTCGAAATTGTTCTATG |
| GB209636 | Perfect | AAAATACCC | 9 | 2 | 18 | TCTTTCCAAATACATTACCCTGC | CTTTTGGGTTTGGGGTTTTTA |
| GB209671 | Perfect | TCCGTTGCGG | 10 | 2 | 20 | TTGATTTGAGATTCTCTGTTTCTTT | CGGGGTGAACATAAATCACAC |
| GB209672 | Perfect | TA | 2 | 11 | 22 | TGACAAAATTGCCATGTCTGA | CTTGCAATTGATCCTACATACG |
| GB209698 | Perfect | TA | 2 | 20 | 40 | GAAAATTCTACACCAAAAACTTGT | AAGGGAAAGAAGGGCACAATA |
| GB209706 | Perfect | GAGAGCAAA | 9 | 2 | 18 | TGCTTTATTCATAGAAAAATGGG | CTACCCAAAATCCCGAATCTC |
| GB209709 | Perfect | AATCTTTTG | 9 | 2 | 18 | ATAGCGGTACCGGGTAATTTG | GAGCATTCCTTGCTCTCCATA |
| GB209765 | Perfect | ATCCCC | 6 | 3 | 18 | GAATTTTTGCCTCCATCCC | TTTTTGCAACGTCTTTTTGGT |
| GB209769 | Perfect | TA | 2 | 10 | 20 | TCTCTCTCTCTCTCTCTCTCTCTCTC | GCACAAGCAAATTGGTTTCAT |
| GB209774 | Perfect | AT | 2 | 13 | 26 | TGCAAGATAACCATCATGCTAAC | TGTATCGACGCACGAAATGTA |
| GB209794 | Perfect | CAT | 3 | 10 | 30 | CAAGCCTTTAAATGTTGTTTTACCT | AGTGTTTGTGGGTTGTTCAGG |
| GB209825 | Perfect | TATTTATTT | 9 | 2 | 18 | CTGGATCAAATGTTTTACAGGA | ACATTTGTCCTTTGTTCCAGC |
| GB209836 | Perfect | TAGCGAGTAG | 10 | 2 | 20 | TTGTTCTTGCTAGAAACCCAGA | AGGACTGACGAAGGAGACACA |
| GB209844 | Perfect | AAAAAAAAT | 9 | 2 | 18 | AAACCCACCATATGAAAATA | TTATCTTGCTCCAGGGAGGTT |
| GB209867 | Perfect | TTTCATCAA | 9 | 2 | 18 | AAAATCTTCAATTTCTGATGTCCA | AGCATTTGATAGGCCGTAGGT |
| GB209896 | Perfect | CTTTTGTTG | 9 | 2 | 18 | TTGTACGGTTATCTCCCCTGTT | ACACGTTTAACCCACTCCTCC |
| GB209906 | Perfect | ATTCAAAAAT | 10 | 2 | 20 | TCCCATATGTGCTTGAGCATT | ACAGATGTGGTGGGGATATTG |
| GB209910 | Perfect | AT | 2 | 22 | 44 | CAACCAAATTAGGTAAAAACACGA | TCGATTTCGAGCAGATTGATAA |
| GB209947 | Perfect | TTTATT | 6 | 3 | 18 | ATGTTTCTCTTACATTGTGTGAA | TCATTTTTCATTGGCTTCTCG |
| GB209960 | Perfect | CCAAAAACC | 9 | 2 | 18 | TTCCCAACCAATCGTATTGAA | AGCTCCAGAGCGAGTAAGACC |
| GB210002 | Perfect | TTTTACTAT | 9 | 3 | 27 | TTCTACTATTGTATTCATGTATTCCT | AATATGTCTCCCTTGCGCTCT |
| GB210032 | Perfect | AT | 2 | 11 | 22 | TGGATGAGAAAATGTGATGATGA | GTGTGTGGAAAGGCAATCAAT |
| GB210056 | Perfect | TGCGGTTTT | 9 | 2 | 18 | CATCCAAATACATCCAAACCAA | GAGGTGATCATATCCGAACCA |
| GB210064 | Perfect | CTAGTATACA | 10 | 2 | 20 | CCTGCTCTCCAAGATTATGACA | CCAAACCACAACTCCCTCTTT |
| GB210082 | Perfect | ATAGCTTATA | 10 | 2 | 20 | TGGTTCTGTTTGGTAAAAATAACTGA | TGGTTGAACCGCTAATTTTGT |
| GB210102 | Perfect | TGT | 3 | 10 | 30 | TCTGTCTTTAGAATTGGAATCA | ATTGGTATCAGGAGCTGGGTT |
| GB210105 | Perfect | TGT | 3 | 6 | 18 | CATGAAGTTGAGGATGTTGTGG | AAGGGCACTTTTGTCCTTTTC |
| GB210403 | Perfect | ATC | 3 | 8 | 24 | CAACCCTTCACCTTCTCCATT | CTGCTGCTTCTTGTCTTTTGG |
| GB210413 | Perfect | AT | 2 | 9 | 18 | TGAAAAGCAACAGCGGATTTA | AGTTGTACTCGCGGTTTTGAC |
| GB210423 | Perfect | CTAT | 4 | 7 | 28 | TTTTGGACTTGAGTCCCATTG | AGAGAGAAAAGGGTGAGACGG |
| GB210429 | Perfect | CAT | 3 | 7 | 21 | TCGGAGTTTTAGGGTTTCTCA | GGAGATTCAGTGACGTTGAGG |
| GB210432 | Perfect | AC | 2 | 13 | 26 | ACACCACCACCTCAACACAAC | TTCCACGATACAAGGAAGTGG |
| GB210445 | Perfect | CA | 2 | 17 | 34 | AACACACACACACACACACACA | AACTCACAAGGCGAGAGAATG |
| GB210450 | Perfect | GT | 2 | 13 | 26 | TCCAACAGTGAATTTTGTGAAA | CCGTAACTGTCGATGCTTGTT |
| GB210453 | Perfect | GA | 2 | 15 | 30 | TGCATTTGTGTGTGTGTGTGT | CAAGGAAGAAATCTGTCTGCG |
| GB210461 | Perfect | TG | 2 | 11 | 22 | TTTTCTTGGAACCTCATTGTTTC | ATCATGTCCTGCACCATTGTT |
| GB210464 | Perfect | CA | 2 | 9 | 18 | GGAAAATGCACACACACACG | TAAGGGAGGGCGTTGTAATTT |
| GB210465 | Perfect | AC | 2 | 14 | 28 | CACGTCGCCAGTTTGTACC | GCTTGTGTGTGTGTGTGTGTG |
| GB210467 | Perfect | CG | 2 | 10 | 20 | CACACGTAACGCACATACACTC | AGGTGAGTAGCGTGTAACGGA |
| GB210468 | Perfect | CA | 2 | 13 | 26 | ACACGTGTGTCAGTGCG | GACCATCGTCCAGGTGAGTAG |
| GB210488 | Perfect | CTAT | 4 | 13 | 52 | TGAAGTCCGGTGTCTATCAGC | CAAAAACGATTTCCCACTTCA |
| GB210493 | Perfect | ATAC | 4 | 7 | 28 | CACACACACCACACACACAAA | TTGAGATGTGCACAAGGACAC |
| GB210496 | Perfect | CACACACAC | 9 | 2 | 18 | AATAGCATTCACAAACGCACA | TGATTGAGATGTGCACAAGGA |
| GB210501 | Perfect | TG | 2 | 10 | 20 | ATTGTCGCTGTCGCCTGT | TACCTCACTCCTCACGCAACT |
| GB210504 | Perfect | CAC | 3 | 6 | 18 | AAACAACCGCCAAACTGAC | GGTTCTAGCCGTGGTTTAGGT |
| GB210505 | Perfect | AC | 2 | 11 | 22 | CTTTCAAAGCTGTTTATCCAAA | TTCTCTAACAATCCTGCCCAA |
| GB210506 | Perfect | GA | 2 | 17 | 34 | ACACAAAGGAGCGAATGAGAT | CTCTAACAATCCTGCCCAATG |
| GB210507 | Perfect | AC | 2 | 12 | 24 | CACACAATTTTCATACGCGCT | GGATGCAGAATATCCGTTCCT |
| GB210515 | Perfect | GTGCGTGTGC | 10 | 2 | 20 | TTGTGTGTGTGTGTGTGTGTG | GAGGAACGTGAAACGTGAAAC |
| GB210518 | Perfect | CA | 2 | 12 | 24 | CCACCCCCATAACCACTGT | TCTGGATCATTGTGGAGGTTC |
| GB210526 | Perfect | GAT | 3 | 9 | 27 | TGTGAGCTGGTGAATTGAGTG | TTCCCAAATAACCAAAGGAGG |
| GB210530 | Perfect | CAT | 3 | 11 | 33 | AAGCCATTAAATGTTGTTTTACCT | TCTTGACAGGTTTCTTGAGGG |
| GB210534 | Perfect | TCA | 3 | 6 | 18 | TGTTTCATCAAACTCTGTTTCTCA | TTAGGATGAACCCTAGCAGCA |
| GB210539 | Perfect | ATC | 3 | 9 | 27 | AATTCTGCAATTCCACTCTGA | GGGGGAAGACATATTGGAGAG |
| GB210540 | Perfect | TTC | 3 | 7 | 21 | TGCAGAGTCTAGAAGCAGAGTT | TGTTTCAAAGCCATGTCCATT |
| GB210541 | Perfect | TTCT | 4 | 5 | 20 | TTTTCAGGATTCTCTTCTTTCTCAA | TGTTTCAAAGCCATGTCCATT |
| GB210546 | Perfect | ATG | 3 | 8 | 24 | GCGTCTTCCAACAGAGTTTTG | CAGCTCAAGTCACTTCGCTTC |
| GB210547 | Perfect | ATCT | 4 | 12 | 48 | GAGATTCATTTCCACAAAGACT | GGATTCAGTCGTTGGATCGTA |
| GB210555 | Perfect | CCCTTTCTT | 9 | 2 | 18 | AAGGAATGCACATCTTCCTGA | AAAGCAGAGTGGAATTGGGAT |
| GB210566 | Perfect | TCA | 3 | 6 | 18 | TTGTTTCATCAAACTCTGTTTCTCA | GGTGAAGGTTGGGTAGGGTTA |
| GB210575 | Perfect | TG | 2 | 16 | 32 | GTCGCGTGTATGTGTGTGC | GAAGGTCTTCATTAGCCGAGG |
| GB210576 | Perfect | GA | 2 | 12 | 24 | TGTGTGTGTGTGTGTGTGTGA | GAAGGTCTTCATTAGCCGAGG |
| GB210582 | Perfect | CA | 2 | 10 | 20 | AGCACACACACGCACACAC | GCGTTAGTCAGTCGGTGACAT |
| GB210584 | Perfect | GT | 2 | 15 | 30 | GGCGCACTGTGCTCTATATC | GTAGACCGTGTTCGCGTAATG |
| GB210588 | Perfect | GT | 2 | 12 | 24 | AAACTATCAGATATTGTTGCCTGGT | AAGCTTACTCAGCTAAAGTGAT |
| GB210602 | Perfect | AC | 2 | 10 | 20 | CGAAATATGTCAGCAAGCACA | GTTTCCTCTGGTGATGTTCCA |
| GB210603 | Perfect | AG | 2 | 15 | 30 | CACAAACACACACACACACACA | ACAGTCAATGTGGCAGCTCTT |
| GB210605 | Perfect | GT | 2 | 11 | 22 | GCTTAATGCCCAATGGCTG | AAAATAGCGCGACTCACTTCA |
| GB210609 | Perfect | GT | 2 | 9 | 18 | TCAGGAATGCATACGAGAGAGA | CACTCAAAGGCAAAAACCAAA |
| GB210610 | Perfect | TG | 2 | 25 | 50 | CGCCGTATGAACGTATGAGTC | CTTCCTGCAAATAATCGGGAC |
| GB210612 | Perfect | CA | 2 | 17 | 34 | AATGGAAATATTGAATACGC | TCAACCAGATTGCTTGGATTC |
| GB210613 | Perfect | AC | 2 | 18 | 36 | TGCTGTTGTTATCCTAAATCCC | ATTCAATTCTGGACGCACATC |
| GB210614 | Perfect | TG | 2 | 14 | 28 | ATGACATATCATGTCCACCCA | CATGAACTTGCAAATAAATGTCA |
| GB210615 | Perfect | GA | 2 | 9 | 18 | CCCATAATGTGTGTGTGTGTGT | CATGAACTTGCAAATAAATGTCA |
| GB210621 | Perfect | CA | 2 | 9 | 18 | CACACGCCTCTACCCTTTTT | TGCTCTGAGAGAAGCCATGAT |
| GB210626 | Perfect | TCA | 3 | 10 | 30 | GGGTTCAATAATACAAAGCCAGA | ACGAAGAAGATGACGCTGAGA |
| GB210629 | Perfect | GCT | 3 | 6 | 18 | GAGCATGTAGCAGTAGCTGATGT | AAGAATAACATGGGAACGCCT |
| GB210631 | Perfect | TCA | 3 | 10 | 30 | GCAACAACACTAACCCTAGCAA | GAAGGAGGATGTGTGGATGAA |
| GB210636 | Perfect | CAT | 3 | 9 | 27 | CCAAGCCTTTAAATGTTGTTTT | AGTGTTTGTGGGTTGTTCAGG |
| GB210639 | Perfect | TCTA | 4 | 7 | 28 | GGCAAACCGTACTTCCTTACG | TCAGTCGGAATCAGGAGAAGA |
| GB210646 | Perfect | ATC | 3 | 7 | 21 | TTGCCATTGTGAATTTTT | ATTAATTATCGCTAACGGCGG |
| GB210653 | Perfect | TATC | 4 | 9 | 36 | TGTACTCGCGGTTTTGACTACT | CGACCACCACCAGAGAATCTA |
| GB210656 | Perfect | TG | 2 | 9 | 18 | ATACGTTCTTTATTGTCGCGG | AGGATAGCCTCGAACAGAACC |
| GB210671 | Perfect | TGGA | 4 | 5 | 20 | GACTCCTCACTCAGCCTTTCA | ACTCCCATTAGTGTTGGGACC |
| GB210673 | Perfect | ATTGTA | 6 | 3 | 18 | GGATGGATGTGCTTGTTTGTT | CATTAGTGTTGGGACCTGCAT |
| GB210675 | Perfect | TG | 2 | 20 | 40 | GGAATGTTGTGTGGTGCG | TTCTCATGTCTTACGGTGATGG |
| GB210676 | Perfect | GA | 2 | 11 | 22 | GTTTCGTCGTGCGATATGACT | GTGATCGGAGTCACACGAGAT |
| GB210689 | Perfect | GC | 2 | 10 | 20 | CTACCCCAAAAGTAACCGAGC | TACTCGTAGCGCAAGTGTGTG |
| GB210691 | Perfect | TG | 2 | 19 | 38 | GGTATGTGTGCCTGTGTTGC | CGGACCAGGAAGAAAGAATTT |
| GB210692 | Perfect | TG | 2 | 10 | 20 | GTGGGCGGGGGTAAATTCTTT | AAGCTTCTGTGCTGTGCCC |
| GB210694 | Perfect | GT | 2 | 12 | 24 | GTCTGTCTGTATGTGTGTGCGT | AGCTTACCTCGCAAGCACG |
| GB210703 | Perfect | GT | 2 | 12 | 24 | CGACATTTTCCAAAAGGGTCTA | CCGACTCGTGAGACAGGTTAC |
| GB210705 | Perfect | AC | 2 | 21 | 42 | GTCACGCTTAACCCTCTCACA | GTGGACATTCGCAGATGACTC |
| GB210707 | Perfect | TC | 2 | 13 | 26 | TTGTTCTCTCTCTTCTCAACCCA | AACATCATCCTCATCAATCGG |
| GB210708 | Perfect | CA | 2 | 11 | 22 | TCTCAACCCATTCTCTCTCTCTC | AACATCATCCTCATCAATCGG |
| GB210709 | Perfect | TC | 2 | 12 | 24 | TTCTCTCTCTCACTGTTCTCTCTCTT | AACATCATCCTCATCAATCGG |
| GB210710 | Perfect | AC | 2 | 13 | 26 | TCTCTCTCTCTCTCTCACAAAGACAA | AACATCATCCTCATCAATCGG |
| GB210711 | Perfect | CTCA | 4 | 7 | 28 | CTCTCTTCTCTCCACCCCCTA | AACATCATCCTCATCAATCGG |
| GB210715 | Perfect | GT | 2 | 16 | 32 | AAGCAAAAGGAGACTTTCACAA | GGCTTGGTCTGGACCTCTATC |
| GB210717 | Perfect | GT | 2 | 11 | 22 | AAATATCTCGAGCTTGGAGCAG | TTTCCACAGAATTTGTCAGGG |
| GB210719 | Perfect | AC | 2 | 14 | 28 | TCCGTCTTTCTCTTTCTCTCTCA | GTGTATGATTTCCGCCCCT |
| GB210720 | Perfect | AG | 2 | 9 | 18 | ACACACACACACACACACACG | GTGTATGATTTCCGCCCCT |
| GB210723 | Perfect | CA | 2 | 13 | 26 | ACAAATGGGATGTTTTCCTCC | GTGTCTGACCTTGACCTCCAA |
| GB210729 | Perfect | CA | 2 | 9 | 18 | CCCTAAACACACACACGCAC | TTGGATTGCGGTCATTTAGAC |
| GB210733 | Perfect | TG | 2 | 10 | 20 | CGTGAGAGTAGCACCATACCC | AATTAACACGACACGCAGACA |
| GB210734 | Perfect | TG | 2 | 10 | 20 | GAGTAGCACCATACCCTTGTG | CAAATTAACACGACACGCAGA |
| GB210738 | Perfect | TGCCTACCAA | 10 | 2 | 20 | ATGCGTGTTGGTGGACG | CATGAAACATTTGCGATTCCT |
| GB210744 | Perfect | GT | 2 | 15 | 30 | CAATCTTATTGTGTGGGTGGG | AATAAGTCAACGCCAAGGGTC |
| GB210745 | Perfect | GA | 2 | 10 | 20 | CGTGTGTGTGTGTGTGTGTGT | AATAAGTCAACGCCAAGGGTC |
| GB210748 | Perfect | AC | 2 | 19 | 38 | TCTCTCTCTCTCTCTCACTCACACA | TAAACGGGGAGATAGGTGGAG |
| GB210749 | Perfect | AC | 2 | 14 | 28 | CGTCCATTAGTCACTCACACA | CAGACATGAGATGCGATGAGA |
| GB210753 | Perfect | GA | 2 | 15 | 30 | TGAGAGAGAGAAAGAGAGAGGG | CGTTGTAAGCCTCCTACGAAA |
| GB210754 | Perfect | CA | 2 | 9 | 18 | ACATTTTGAAAGTAGCGTGCC | CACAAAGTAGCGCCCTATGAC |
| GB210757 | Perfect | TCTA | 4 | 9 | 36 | CACACACGCACAGGACAAC | TCCGGGGCAATTTATATCTCT |
| GB210762 | Perfect | CAT | 3 | 13 | 39 | TTCTCCCATCTCCTTCCATTT | CTACCTTCATTGCCAATTCCA |
| GB210781 | Perfect | CA | 2 | 10 | 20 | AAACACGCACACACACACG | AAGCTTCCATTTTGCACAGG |
| GB210783 | Perfect | TG | 2 | 24 | 48 | TTTTTGCTTGTGCTTGTTTTT | ATCCCGTGGTAGTGCTTACCT |
| GB210790 | Perfect | CA | 2 | 14 | 28 | TCGTCTTGCGAAGCTAGTGAT | GCTCTGTCTGCTTTATCCCCT |
| GB210792 | Perfect | GT | 2 | 13 | 26 | CATCAAAGACCAACACCAACA | AGTGGTGATACAAGCAGGTCG |
| GB210800 | Perfect | AC | 2 | 16 | 32 | CACATATGGATCGAGTCGTGC | CATTTTGTCTTGAACGTCGGT |
| GB210806 | Perfect | GT | 2 | 15 | 30 | GACTTGGAGCAAAAGCAAAGG | GCAGTTCCAGTTCCATAATGC |
| GB210810 | Perfect | GT | 2 | 16 | 32 | ATGCCGCCGGTCAGTAAAC | TGTTAGCCTTGCTGTTAGCGT |
| GB210819 | Perfect | CA | 2 | 20 | 40 | TTTGATCTCTCTCTCTTCTCTCTCTC | TTTGGCTTTTTGTCTGGTCAT |
| GB210824 | Perfect | GT | 2 | 9 | 18 | GTGACGAGTGTGTGGTGTGTT | AGCACGGAGAGAGACAAGTCA |
| GB210825 | Perfect | TG | 2 | 16 | 32 | TCGAGTCGGATCGCTCT | AACCCACGTAATGGAATGACA |
| GB210826 | Perfect | ACTG | 4 | 6 | 24 | GCCCCAGAGGATGTAGGTCT | AACCCACGTAATGGAATGACA |
| GB210831 | Perfect | TG | 2 | 9 | 18 | CGTTCGTGTGTGTGTGTGTG | ATAGACCGGTGGTAAGCCAAT |
| GB210836 | Perfect | CG | 2 | 9 | 18 | GCACACACACACACACACACA | GTGGCGTGTGGATACAGAGAG |
| GB210840 | Perfect | CA | 2 | 17 | 34 | GGGCATGAAAAGGAAGAGG | TCACATCTCGCAGTTAGCAGA |
| GB210853 | Perfect | GT | 2 | 19 | 38 | ACGGAGCAATCACAACTGC | ACGTGGACAGACGTGGATAAG |
| GB210856 | Perfect | AACAACAAA | 9 | 2 | 18 | ATCCACGTCTGTCCACGTCTA | TGTCGAAGGAAGGGAGATGTA |
| GB210857 | Perfect | GT | 2 | 12 | 24 | GGAAGTGGAAGGAAAAGGAGA | GGGTGTATCCAGCGACTTACA |
| GB210858 | Perfect | GA | 2 | 25 | 50 | AGTGTGTGTGTGTGTGTGTGTGT | GGGTGTATCCAGCGACTTACA |
| GB210868 | Perfect | CA | 2 | 10 | 20 | TTCTGACACACACACTCTGGC | TTCTCGAGCGACTCGGTGT |
| GB210869 | Perfect | CA | 2 | 12 | 24 | GCACACACACACACACACACA | TTCTCGAGCGACTCGGTGT |
| GB210873 | Perfect | GT | 2 | 14 | 28 | TGTGTGTGTGTGTGTGTGTGC | ACTGTGATGTGAGTCATGCCA |
| GB210879 | Perfect | AC | 2 | 9 | 18 | CATGGGGACTTTAAAGGGTGT | CGTGTACCGGGTGTATTATCG |
| GB210883 | Perfect | ACATACACAC | 10 | 2 | 20 | GGGTGTAAGTTATCGGGGAAA | CGTGTACCGGGTGTATTATCG |
| GB210884 | Perfect | GT | 2 | 25 | 50 | ATTGTGTGGCTAATGAGGACG | TTCAAAAATGACCTCGAAACG |
| GB210885 | Perfect | GCTTTAAGAT | 10 | 2 | 20 | GTAAGGATCATAGCGTGTCCG | AAGCTTACAGCAGGCAAGATG |
| GB210886 | Perfect | CA | 2 | 16 | 32 | CTCTAACCCTTTCACCCCTCC | TGGTCCTCATACCTGGAACAG |
| GB210890 | Perfect | CA | 2 | 17 | 34 | TGCCGGACAAAGCGAA | GAAGAGGAAGGGATTGAGCC |
| GB210891 | Perfect | TG | 2 | 13 | 26 | CGCACAGCAGATTCGTGACT | CCGTCCTAGCCAATATACCGA |
| GB210896 | Perfect | TTTTTTG | 7 | 3 | 21 | AGTTATAGACTGTATAGTTGCTGTTT | TTTTCGGTTCACGTTTTGAAG |
| GB210899 | Perfect | CA | 2 | 12 | 24 | ACACCCTGGCTTTCTTCCTAA | AAGCTTCCCAGGAGGAACTCT |
| GB210900 | Perfect | TG | 2 | 20 | 40 | TGAAATCCCACAGATAAGAAAGG | TTCACATCCCCCTGTATTCTG |
| GB210901 | Perfect | CA | 2 | 12 | 24 | GCACGTGCAAGTGAGAATA | ATAGGTCTTGCGTGTCTGCAC |
| GB210902 | Perfect | CTG | 3 | 10 | 30 | GCTACTGCTGCTACTGCTGCT | TCCAGAAGGTATACCACGACG |
| GB210905 | Perfect | CTGCTGCTA | 9 | 3 | 27 | TTACTGCTGGGGCTGAG | TCCAGAAGGTATACCACGACG |
| GB210906 | Perfect | TG | 2 | 16 | 32 | AAAAATGGCTAATGGGTTTTT | GCGAAGAGTCAAAGCTCTCAA |
| GB210907 | Perfect | CA | 2 | 14 | 28 | GCCCACACTCACACACACA | CTCCTGACCATTTCCAATCAA |
| GB210918 | Perfect | CT | 2 | 19 | 38 | TCTCTGGAGGGGATTTTGTC | GGGAGCGTGAGTTAGTGAGTG |
| GB210920 | Perfect | AC | 2 | 19 | 38 | GAGACAGCCACTCACACCA | GTGTATAGAGCCGGGGGAATA |
| GB210922 | Perfect | CA | 2 | 10 | 20 | CATATACACACACACGCGCAC | CCATCTTATTTTCCCTCCCAA |
| GB210923 | Perfect | TTGAAG | 6 | 3 | 18 | CCCGAGTCGTTATAACCCTTT | CTAGGTGTTGAACAAGCCGTC |
| GB210926 | Perfect | AGAC | 4 | 10 | 40 | ACGAGAAATAAAAAGGCAGGC | CCCCATCTAATAGTTCACGCA |
| GB210927 | Perfect | AGAT | 4 | 14 | 56 | ACAGACAGACAGACAGACAGAC | CCCCATCTAATAGTTCACGCA |
| GB210929 | Perfect | TATC | 4 | 12 | 48 | TTCTTCCATCAAATTGTTGACCT | CACCGCAACCATTACAGAGTT |
| GB210937 | Perfect | TATC | 4 | 8 | 32 | ATATCAAACGCTGATCCCCTT | GTCGATTGGCAACTTCTTCTG |
| GB210941 | Perfect | GATA | 4 | 12 | 48 | CAACACAACGAGGGCTTTG | CCCAATCGTATCGTGAACAGT |
| GB210943 | Perfect | CTAT | 4 | 10 | 40 | TTTCAAAAATATGCTACTATGGTGA | GCCCACCTCTCTCTGTTTTCT |
| GB210947 | Perfect | TCTA | 4 | 10 | 40 | GTTAGCAGTGAGGCAAGTGGA | TTTTGTTGGTAGCTTGGTTGG |
| GB210959 | Perfect | ATC | 3 | 8 | 24 | CCCCCTTCATTTCTTTAATTGC | TGGACCTGGTTCTTCATCATC |
| GB210960 | Perfect | AGT | 3 | 6 | 18 | AATTAGCTAGCTCTATCAATCATGT | GGACCTGGTTCTTCATCATCA |
| GB210970 | Perfect | CAT | 3 | 9 | 27 | GCTTCGACCTCGACAACCT | GACGCTCCTCTTCCTTCTCAT |
| GB210974 | Perfect | ATG | 3 | 8 | 24 | CGGTTGACAGAGACGAGGATA | AAGACAATATTTACCGGCGCT |
| GB210977 | Perfect | CAT | 3 | 10 | 30 | CTTCGACCTCGACAACCTG | GACGCTCCTCTTCCTTCTCAT |
| GB210979 | Perfect | ATG | 3 | 7 | 21 | CGAGGGATCGAAAGAGAGAAA | CGATTTCGGAGTTTTAGGGTT |
| GB210982 | Perfect | TCA | 3 | 6 | 18 | ACCAAACCAAGACATTGTAACT | TCAGATTCAGAGGATGAGGATG |
| GB210983 | Perfect | ATC | 3 | 6 | 18 | TCAGAAAACTCATCATCATCATC | AGCTTCCAGAATCTTTTCAATG |
| GB210985 | Perfect | TG | 2 | 12 | 24 | TTGGCAATGTCAGTAGCTTTG | GTTTGATCAATTCTCAGGGCA |
| GB210986 | Perfect | GA | 2 | 15 | 30 | CATGCATGTGTGTGTGTGTGT | CAAGGAAGAAATCTGTCTGCG |
| GB210989 | Perfect | TG | 2 | 13 | 26 | CCTTAAATTAATGAAGTCGTGTTTT | GCCATCCTTGGTTGTACAGAA |
| GB210991 | Perfect | CA | 2 | 18 | 36 | TCTCTCTCTCTCTCTCTCTCTCTCTC | GGGTGAGAGTCTGTGTGAGGT |
| GB210992 | Perfect | GC | 2 | 10 | 20 | CTATGGAGCCGTTCATTCA | AGCCACCCGGAACACACACA |
| GB210996 | Perfect | GA | 2 | 15 | 30 | TTTGTGTGTGTGTGTGTGTGTG | GTTTGATCAATTCTCAGGGCA |
| GB211006 | Perfect | CA | 2 | 11 | 22 | CGCACACACATGCACACAC | TATATCTTTCGTCAGTGGCGG |
| GB211009 | Perfect | AC | 2 | 13 | 26 | GTATCGGTGGCGCTCAAAAC | CAAGCGTGTTTGGAAGTTTGT |
| GB211014 | Perfect | TC | 2 | 13 | 26 | CAAAACTCTCTCTCTCTCTCTGT | ATCAACGTCTGAGTCACTGCC |
| GB211020 | Perfect | CA | 2 | 13 | 26 | CCTCCTCGTTGTATACCGTGC | AACTTACCTACGACGCGCT |
| GB211025 | Perfect | CA | 2 | 19 | 38 | TTGCTCTCTCTCTCTCTCTCTCT | TATGGCTGCAATAAAAATCCG |
| GB211026 | Perfect | CA | 2 | 9 | 18 | TGTGAGATATAATTGGATACACGC | AAGCTTCTGTTTTCATACAATG |
| GB211031 | Perfect | ATAG | 4 | 10 | 40 | AATGTGCAAAACATGTCATCCT | CAACGAAAGCAAGAATTTCCA |
| GB211033 | Perfect | ATAG | 4 | 7 | 28 | GGGTTTAGCATCATGAGCTTTT | CGATGATTTTGGAGGCTTTTA |
| GB211036 | Perfect | CCCTTTCTT | 9 | 2 | 18 | TGAATGCACATCTTCCTGATG | GGAGTCAGTCGTTGGATCGTA |
| GB211037 | Perfect | ATCT | 4 | 7 | 28 | AGCAGAGATTCATTTCCACGA | GGAGTCAGTCGTTGGATCGTA |
| GB211039 | Perfect | CCCTTTCTT | 9 | 2 | 18 | TGAATGTACATCTTCCTGATGACT | AAGCAGAGTGGAATTGGGAGT |
| GB211041 | Perfect | TATC | 4 | 9 | 36 | AGTAATGCTCTGATAATGACTTATG | CAAACCACCAACACCGACTAT |
| GB211051 | Perfect | ATCT | 4 | 8 | 32 | AAGCTTCCAGGTGCATGA | CACCCCCTCTCCGATAAACTA |
| GB211054 | Perfect | TC | 2 | 13 | 26 | CAACCAAACAAAATAATCCTTACG | ACTTGCCAGACCGTAGTCAGA |
| GB211055 | Perfect | TCTA | 4 | 5 | 20 | CGTTATTTCAGTTTTCTCTCTCTCTC | ACTTGCCAGACCGTAGTCAGA |
| GB211061 | Perfect | AT | 2 | 10 | 20 | AGCAACAGCGGATTTAATCTT | GCTTACTCGCGGTTTTGACTA |
| GB211097 | Perfect | ACTAATTACA | 10 | 2 | 20 | GCTTCTGTTAACTCTAGTTCATTCT | CCAACTTCGGTTTTGTGGTTA |
| GB211141 | Imperfect | TTTTCT | 6 | 3 | 18 | CATTTTCTCATTTTTGGTTCACA | TCCAGTTGGAATTAAGAGCGA |
| GB211155 | Imperfect | GCTATAA | 7 | 3 | 21 | TGAATTACACAGATTGATCATTTGAC | ATATGCAAAACTCTAAAACGAA |
| GB211159 | Imperfect | TATGCA | 6 | 3 | 18 | TTCAGGTTTTCATATACAAGTTAGC | GGGATAAAATATTATTCAGCAACGA |
| GB211169 | Imperfect | TCCTTT | 6 | 3 | 18 | GGACTTGGATGTGGAGGTCTT | GAGACCATCGAGGATATGCAA |
| GB211176 | Imperfect | TAATTT | 6 | 3 | 18 | GGATGAGGTGATTACCCATTGT | ACTTGAGCATCAGCACCAGAT |
| GB211179 | Imperfect | TATACA | 6 | 3 | 18 | TCAAATGAAAGAACTTAAGGAA | CGATTTGCCTATAATTTCCCA |
| GB211190 | Imperfect | TTTAAAT | 7 | 3 | 21 | TGGTTTATGATTTTCATGTTTGT | CCAGGACTAGCAAGAATGCTG |
| GB211200 | Imperfect | ATTTTTT | 7 | 3 | 21 | TCCTTCTCATGCTTATCACCG | TTCCGTCATTTGAATTCCATC |
| GB211201 | Imperfect | AAATT | 5 | 4 | 20 | TTGACATTAATTACAATAAGCAGA | TGACGTTGTTGTTTCACCAAA |
| GB211207 | Imperfect | ACAAGG | 6 | 3 | 18 | AAGGCGTAAGATTTCTGGACAA | GATTGTTGACATTTGGTTGGG |
| GB211210 | Imperfect | TCA | 3 | 6 | 18 | GGAGATCACTAATCAAGCAAGG | TTTAGTACAAGCCATGCACACC |
| GB211211 | Imperfect | ATATCA | 6 | 3 | 18 | GGTGTGCATGGCTTGTACTAAA | TTTTCCTCTTATAATTTTGAACGGT |
| GB211230 | Imperfect | TCTATAT | 7 | 3 | 21 | TTCCACTCTTTGTGAATCATCTC | AAGATAAATGGTGGGTGGGAC |
| GB211234 | Imperfect | AGTTTTA | 7 | 3 | 21 | TGTCACCTGCTGCTTGTCATA | ACATCACGGCTAGCAGGACTA |
| GB211239 | Imperfect | TTAAAA | 6 | 3 | 18 | CAGAAACTCACATTCCATTCTCA | TGTGTTTGTATCCGTGCTTCA |
| GB211254 | Imperfect | ATGTTT | 6 | 3 | 18 | TTAGAATCCCAATTCCAACTCC | GACACTGGGTAGTATGCTCGC |
| GB211269 | Imperfect | ATATAA | 6 | 3 | 18 | GTTCCAAACAGCTCCAAACAC | CATTGCTGCTTTTGCTTGAGT |
| GB211294 | Imperfect | TTTGTT | 6 | 3 | 18 | TGCATATTAAAGGTTTTGATCCG | ATGCTAGCAGCCATATTGACG |
| GB211295 | Imperfect | TTGATTAA | 8 | 3 | 24 | TTGACTGCATCGATATAATTTGC | CTGCGATTTAACACAACAGTCAT |
| GB211299 | Imperfect | TTTAAG | 6 | 3 | 18 | CTCTGTCATTCTTTCTTTTCTTTCA | GGACTCACGTCCATCTGACAC |
| GB211304 | Imperfect | TTGTTT | 6 | 3 | 18 | CACACGATCTTGAGTTAGCCG | CTTTGCCATGAGACTCGACAT |
| GB211308 | Imperfect | TTCTTT | 6 | 3 | 18 | CCAAACACATCTAAAAATTGGTCA | GGCAAAGGGACCCAAAAGTAT |
| GB211313 | Imperfect | ATTTTA | 6 | 3 | 18 | AAACATGTGATATTCACGATTGTAG | TCGTCAATGGATATGAAGCAA |
| GB211317 | Imperfect | GTTTTGA | 7 | 3 | 21 | CAACAAATATGAACTTAAAACCAACA | ACCGAACCAAACCATTTGAAC |
| GB211318 | Imperfect | TAATTG | 6 | 3 | 18 | TTGATTTCTACTGCATTTGTGT | TGCTCAAACTTCAACCCTCAC |
| GB211324 | Imperfect | ATTTTA | 6 | 3 | 18 | CAGACCAAATTTCAGTGGCAT | CGGTAACGAAAGTTTACGCAG |
| GB211341 | Imperfect | ATCTAT | 6 | 3 | 18 | TGACTTCCCTCCTAATCTCCC | CAGTCCAGTAAATCGAATCGTG |
| GB211349 | Imperfect | AG | 2 | 10 | 20 | TTGAAGTTGTTGAGTTTATGTTGGA | GCCTTGTCCCGGTTATACACT |
| GB211369 | Imperfect | TAATAG | 6 | 3 | 18 | TGAAACAATTAACAAATGGAGTGG | GTTGATCCAACCCGACCTATT |
| GB211377 | Imperfect | TATGCC | 6 | 3 | 18 | TTCACTCAGTTTGGTTGTAACTTG | GGCATCCGTTGATTTTCTGTA |
| GB211381 | Imperfect | AAGTTTGATT | 10 | 3 | 30 | TCCCCCTTTTTCATCCATTAT | GGGGAATTTAGCATTTGCTTC |
| GB211385 | Imperfect | ACTTCG | 6 | 3 | 18 | GCAAAATATTCAAGGAAAGCG | TCCTTCGATCACTCGTTTCTC |
| GB211386 | Imperfect | ATTCAA | 6 | 3 | 18 | CAACGGCTAAATCAGAAGATAAGA | TCGGGACATAGTGCTTTTGTT |
| GB211392 | Imperfect | ATTTCT | 6 | 3 | 18 | ATACACACATTGTGGGACAAC | TTTCATAAGCTCTCCCGAACA |
| GB211393 | Imperfect | AAAATA | 6 | 3 | 18 | TCCCATTAAGATGACATTGGC | TTTGTTTTGGTTTTTACTAGGGTTG |
| GB211398 | Imperfect | AATACAT | 7 | 3 | 21 | TTGATTACTCTCTTTGTCATTGGC | GAAAACACAAAATGCAGCACC |
| GB211400 | Imperfect | TACAAA | 6 | 3 | 18 | CCGGGGAACTATAGTGTATAATC | TGGTAGTACGCAGAGTCAGGC |
| GB211404 | Imperfect | AAGTGACC | 8 | 3 | 24 | GAAAGAAAGGAGTGAAATATGTGATG | TCTCAAGTGGATTTGGTTTGG |
| GB211408 | Imperfect | CGACA | 5 | 4 | 20 | CCTATGAAGCACGGACTCTGA | GCTAGTTTGACAAAACACGGG |
| GB211409 | Imperfect | CGACAC | 6 | 3 | 18 | CTCCGACACCGCTAATGTAAA | GCTAGTTTGACAAAACACGGG |
| GB211418 | Imperfect | AAAAAT | 6 | 3 | 18 | CCAAAAAGAAATTTACATCAAAAGAC | CGTAAACCGAATCAATCCAAA |
| GB211426 | Imperfect | TAATAG | 6 | 3 | 18 | CACAATATATCGAGGTTTACAACA | TGATCGCACACAACTTCAATC |
| GB211427 | Imperfect | TTCTTT | 6 | 3 | 18 | CCAAACACATCTAAAAATTGGTCA | TAATTGACTATGCTGGCTGGC |
| GB211433 | Imperfect | TTTCTT | 6 | 3 | 18 | TGCATTGTAAGTTCGATTTTAGTCA | AGATTGGATGTCATGGTCAGAA |
| GB211443 | Imperfect | ATAAGG | 6 | 3 | 18 | CAAACTATGGTCATGTCAAACACC | GTGTATGGTTCCCCATCTCAA |
| GB211461 | Imperfect | ATTCTT | 6 | 3 | 18 | CCTCTGATAGTATATCAAGTGTTCCT | AGGGTGCTGAGAGAGAGAAGG |
| GB211469 | Imperfect | TAATCAAACT | 10 | 3 | 30 | CATCCTTGAACTTGAGTTTTCA | TATTTCGCATTCCGCTGATAC |
| GB211483 | Imperfect | ATTACTAA | 8 | 3 | 24 | GCATGACTAATTAATATGAACAAA | GGGGTTTCAGCTGCTATTCTC |
| GB211494 | Imperfect | ATA | 3 | 6 | 18 | GGAACTCATCTACTAAATCAAAAGAA | GGAGCACTGTTTAGCATGACC |
| GB211506 | Imperfect | ACTTCG | 6 | 3 | 18 | TTGTGGACTTAGTAAAATTTCATGT | CCCCTTGCTTAATCACTGACA |
| GB211537 | Imperfect | TTTATTT | 7 | 3 | 21 | GGTAAATTATCCCTTTGATTTT | CCGTGCGTTGATTGTTTAGTT |
| GB211543 | Imperfect | CCGCT | 5 | 4 | 20 | TAGCTGTTCCGCTCCGA | CAATGAGATATGGCCTGGAAA |
| GB211544 | Imperfect | TTAGAA | 6 | 3 | 18 | TCCATTGAAAAAGGGAATTTTG | GGGAGGAAAAACATGTCTGCT |
| GB211549 | Imperfect | AAATAAA | 7 | 3 | 21 | ATACATTAGGTTTATCGTCTAGATT | GTTGTTGGACGAGAAAGTGGA |
| GB211553 | Imperfect | GAG | 3 | 7 | 21 | AGGATGATAGCCCCTATCCAA | GGTTTAGTCCATGTGATGCGT |
| GB211561 | Imperfect | ACTGCA | 6 | 3 | 18 | TAACCACATTGAATCCGCAAT | ATAATGGAGCTGGTTGTGGTG |
| GB211562 | Imperfect | TAA | 3 | 6 | 18 | CACATGCCCCAGCTAAATATAA | CTTTCTTGCTTCATCTGCCAC |
| GB211573 | Imperfect | AAGAAC | 6 | 3 | 18 | GGTTCCTGTCAAAAAGGGAAA | AGCAGTGCTAACTGGTCCAAA |
| GB211576 | Imperfect | TTATAG | 6 | 3 | 18 | TGAAAAGACACTTTAGGAACTGAAGA | TTCGTCAAAAAGGACCTTCAA |
| GB211579 | Imperfect | AAAGTG | 6 | 3 | 18 | AAGGGACATTTAAGAGAAATATAAGT | TCTTCTGTTTGTCTTGGCCTT |
| GB211588 | Imperfect | CAAAATT | 7 | 3 | 21 | ACCGACATATACTCCGGTGG | GGGTGCTTTTACCCTTCAAAC |
| GB211591 | Imperfect | AATCAC | 6 | 3 | 18 | ATATCATGTTTCACCGTCGGG | TAATCCGGTTGATTCATCCAT |
| GB211604 | Imperfect | AAAAATA | 7 | 3 | 21 | CCAATGAGGAGAATGAGTGATG | TTCCACTCGTGTGTGTGTCTC |
| GB211608 | Imperfect | TTAATT | 6 | 3 | 18 | CCACTAAACCAACTAACGATTGG | AAGGGTCCGTGCTTCATAGTT |
| GB211627 | Imperfect | GTAAAA | 6 | 3 | 18 | CAAATTCGAAATGGTAGGAACA | GATCCGCAGGAACTTCTTCTT |
| GB211629 | Imperfect | AATTAAA | 7 | 3 | 21 | GGAATTTCCTGCGGATTTTAT | GGAGTGATTTTGGGGATTGTT |
| GB211633 | Imperfect | ATCCAT | 6 | 3 | 18 | TGGAATTCAGTTTGATCGATGT | CAAGTGAATCATTCTGAGCCAA |
| GB211641 | Imperfect | TAAAAT | 6 | 3 | 18 | ATTATTATCAGATTAAGTCCTGTAGA | TTTTGACGGCATGAAATTAGG |
| GB211663 | Imperfect | TCTCTA | 6 | 3 | 18 | AAGAGTTGAATACAAACCTCTCATTC | GTTCGGTTTAATCGGTTTGGT |
| GB211664 | Imperfect | CCCGAA | 6 | 3 | 18 | GTTTGTCCGGTTCAGAGGTTT | AAAACGCATTCAAATGTACGG |
| GB211672 | Imperfect | ATAAAT | 6 | 3 | 18 | TGTAACTATACATGTCTTGCAGATG | AGAGCCAAACAGCCGAATAAT |
| GB211675 | Imperfect | TAT | 3 | 9 | 27 | GGCAAGAGAAGAATGTGATATGTG | GTTCGAAAATGAAAGCACCAA |
| GB211679 | Imperfect | TCACAG | 6 | 3 | 18 | TCATTGACATTGTGCCAACTC | GCTCAAGGTGACTTTGTTGGA |
| GB211689 | Imperfect | CAGAAT | 6 | 3 | 18 | AGCATCAGAAGCAAAAAGAATTT | AGGTGCCAGCTGGAATTTACT |
| GB211691 | Imperfect | ATTCTT | 6 | 3 | 18 | CCTCTGATAGTATATCAAGTGTTCCT | GCAGCGGATAAAATCACAAAA |
| GB211707 | Imperfect | AATAAC | 6 | 3 | 18 | TCCCACAAAGTATACAAAAGGT | TGGATGATAGTTTGTTCTTGTTTGA |
| GB211710 | Imperfect | CATATA | 6 | 3 | 18 | TTTTTCTTAGCATTTGTACATTGG | ATGCATGATAAAGCATCCACA |
| GB211713 | Imperfect | TATATTTA | 8 | 3 | 24 | TCCAACCACCATACCTATCATC | GCACCCGTCTGTCGTCTAGTA |
| GB211715 | Imperfect | GCCTGA | 6 | 3 | 18 | AAATTTGGAAAGTTTCGATTC | TGAAAAACATAAAGTTTCCGTCA |
| GB211730 | Imperfect | GATCCT | 6 | 3 | 18 | GAATGTTAAACCTGTCTATGTTCA | TGCATTATTTGGATTCCCTTG |
| GB211731 | Imperfect | CTTTCT | 6 | 3 | 18 | CATGACATTAGATCCTGATCCTG | TGCATTATTTGGATTCCCTTG |
| GB211732 | Imperfect | TTC | 3 | 6 | 18 | TCCTGATCCTGAATCCTTTCTC | ATTCCCTTGATGCAGGTTAGG |
| GB211751 | Imperfect | CTTGAT | 6 | 3 | 18 | TCGTCAGCATTAAGAGTTTCTGA | CCACAACACCACAACAAGATG |
| GB211752 | Imperfect | TGTTGTGG | 8 | 3 | 24 | GAGTTTGGTTGGGGATCATTT | GATAAAACCCCTTCACCTCCA |
| GB211760 | Imperfect | TGGATTT | 7 | 3 | 21 | GAAGTTTGGTGGTGGTTTTGA | GCCACTTGACCTAATCCTTCC |
| GB211764 | Imperfect | ACTAGA | 6 | 3 | 18 | CCAAATGTCTTGCTCATGTTG | AAGCTTCGCAAATGCTTACAA |
| GB211772 | Imperfect | TGGTTCA | 7 | 3 | 21 | GCAAGAACCAAGAAGTGGTGT | ATTCAATTGGTGGTGAAAGCA |
| GB211781 | Imperfect | AAAATAT | 7 | 3 | 21 | GCCAAACGAAGCTAGAATTTAT | CGGATATCCATACCCGCTAA |
| GB211789 | Imperfect | TTTTCCAT | 8 | 3 | 24 | GCAACTTAACCTCATCACCAGA | ATCTTGTGCAAGCATATTGGC |
| GB211796 | Imperfect | TAAAAAAA | 8 | 3 | 24 | AATGCAGTGGTTTTTAACAGCATA | CAGCTATTTTGAATAAGAGGAATGC |
| GB211802 | Imperfect | CAATAA | 6 | 3 | 18 | TCAAATCTTCATAACCATCTAAAGC | TTTGGAGCGTTTGATTACGAC |
| GB211804 | Imperfect | AAACT | 5 | 5 | 25 | TGAACGTCTTTAAATGAACAA | ATTCTTTGAAGGCATGGGACT |
| GB211805 | Imperfect | CCAATT | 6 | 3 | 18 | GGTTGGACTGTTCTCGTCATAA | ACTTCTTCGCCATAGGGTCAT |
| GB211806 | Imperfect | TACAAA | 6 | 3 | 18 | TCAGACACACTTGAAACCCAA | TTCGCCATAGGGTCATATCAG |
| GB211810 | Imperfect | ATC | 3 | 6 | 18 | AAGCAGCAACAAGTTCCAAAT | TTCAAACAACATCCATCCACA |
| GB211812 | Imperfect | TTTTAA | 6 | 3 | 18 | ACAAGGGTTGGTGATTTTAATTT | TTCAAACAACATCCATCCACA |
| GB211814 | Imperfect | TGATAA | 6 | 3 | 18 | TGTGGATGGATGTTGTTTGAA | TTGTGCCCTTAGGCACTTAAA |
| GB211823 | Imperfect | ATGTTT | 6 | 3 | 18 | TTTTTCTTGTACCAAACTTAAGCA | TCAGCAAGAAAATCAGCCACT |
| GB211824 | Imperfect | TAGACA | 6 | 3 | 18 | CGAACCTTGGAATTTGTACTTTG | TTCATTATTCGAACAAGACCCA |
| GB211830 | Imperfect | AAAATTT | 7 | 3 | 21 | AATTTCTGCAATAACATTGACG | CACATGTGGATACAGGTGGATT |
| GB211832 | Imperfect | AATGAA | 6 | 3 | 18 | CATAAGAAAGGAGAAGAACCTCC | TGCCCAAGTAAGATGAAGTGC |
| GB211838 | Imperfect | AAATTC | 6 | 3 | 18 | ATAAACCTTACGTCCCCAACA | GGTTCAAGCTCCTCAACCTCT |
| GB211840 | Imperfect | CAGGCT | 6 | 3 | 18 | ACAGAGGTCCTTCTGATGCTG | AATTGGGCAACATCATGGTTA |
| GB211843 | Imperfect | AAACTC | 6 | 3 | 18 | CGGTTATGAATGAAAAAGGTTCA | TTTCTTGTCTTCCTCTTCCCC |
| GB211847 | Imperfect | ATAATG | 6 | 3 | 18 | AAGTGGACAAAACAGAAGCATGT | CTTTGGTCCTTATTGATGTTTTCA |
| GB211853 | Imperfect | AGTGGA | 6 | 3 | 18 | CCTAACGGGGTTGAGTTTGAT | TTCAGGTTGGGAGGGAGTAAC |
| GB211855 | Imperfect | GT | 2 | 15 | 30 | GCGTCGGAATTTCACAAAAA | GGCAGAACCAGTGAAAACAAA |
| GB211857 | Imperfect | CAAAAA | 6 | 3 | 18 | AATCCATCATGTAGCACCACC | CAATTGGATGCATATATGGGG |
| GB211861 | Imperfect | AATCTT | 6 | 3 | 18 | AAAAATTGAAATGTGTCATCATCA | GGGTCAGATTCGGTATGTTCC |
| GB211865 | Imperfect | ATTTTG | 6 | 3 | 18 | CGCGTGTTTCTCATTTTGATT | TGGTAAAGAGTAGGGATGGGC |
| GB211866 | Imperfect | AGGGTTT | 7 | 3 | 21 | CGTGTTTCTCATTTTGATTTCT | TGGTAAAGAGTAGGGATGGGC |
| GB211867 | Imperfect | CAGTTAA | 7 | 3 | 21 | TTTGATTTTAGGGTTTAGGGTTT | GGTAAAGAGTAGGGATGGGCA |
| GB211869 | Imperfect | TTTGGTTT | 8 | 3 | 24 | TAAATGGTTTGGTTCGGTTCA | TGAATATCTCCAACTCAACACC |
| GB211877 | Imperfect | CAGAAT | 6 | 3 | 18 | AGCATCAGAAGCAAAAAGAATTT | TTTGTGCCAGCTGGAATTTAC |
| GB211878 | Imperfect | TGGAAT | 6 | 3 | 18 | CAAAATGGATGAGTGATAAATTGAA | AAGATGATGACCGTTGCAGAG |
| GB211879 | Imperfect | ATTCTT | 6 | 3 | 18 | ACCTCTGATAGTATATCAAGTGTTCC | TGCTCAAGCACACTAGCAAGA |
| GB211886 | Imperfect | AAATA | 5 | 5 | 25 | TGTGCATAGTTAGTAGTTGATACTTT | TTTTGCTTAATCACAGCCTGC |
| GB211907 | Imperfect | TAGATAAAT | 9 | 3 | 27 | TTGTAATCGTTATGTTAAATGGTT | CCCACCACCGGTTATTATTTC |
| GB211915 | Imperfect | ATAAATAT | 8 | 3 | 24 | TTTTTGGACCAATATGAAGTG | GTTTGCACAAGTTTTCCGTTG |
| GB211921 | Imperfect | CCAAAA | 6 | 3 | 18 | GGTGTACGTGGACCGGATT | CCACTACCCAACTCAATCCAA |
| GB211932 | Imperfect | TTTGTTA | 7 | 3 | 21 | GTCAATCTCCATGAAGCATACG | TCGAAACAAAGCTTGCTCAAT |
| GB211933 | Imperfect | TTTGAA | 6 | 3 | 18 | GCCTAAACATTACAGAGAGGACG | GCACTTCGACTTTGATGGAAA |
| GB211938 | Imperfect | ATTAAAT | 7 | 3 | 21 | TGAATCTTTTTCCCAGCTTCA | AATGCCTGTCGAAAATACAGG |
| GB211940 | Imperfect | TGAATATTTT | 10 | 3 | 30 | TCCACCACATTCTTGGAGAAA | GATCCCCTCTCAATCCAACAT |
| GB211941 | Imperfect | TTTTC | 5 | 4 | 20 | CGAGTGCCTTTTATTAGGACTTA | CCCAAAATTGGTTAAGGGTGT |
| GB211946 | Imperfect | AAAAAT | 6 | 3 | 18 | TGGGATATCCATCACAACAGA | CTTGCACCAGTATGGCTGATT |
| GB211953 | Imperfect | AAATAAA | 7 | 3 | 21 | AAAATAGTGGTGAACGATGCAA | CGTCTTTCAAAATCCAAACGA |
| GB211954 | Imperfect | TCT | 3 | 7 | 21 | GGATTTATTTTGAAGTGATGCGT | CGTCTTTCAAAATCCAAACGA |
| GB211966 | Imperfect | TAGCTTA | 7 | 3 | 21 | ATTGGCTCTGTTTGGTAAGGC | TGAAAATGAAACCACGGAAAT |
| GB211968 | Imperfect | AGTCATC | 7 | 3 | 21 | CCAAACACTTCAATTAGCTTATCA | TGTTTGGTAAAAATAGCGGTTG |
| GB211969 | Imperfect | ATAAGTC | 7 | 3 | 21 | AGCTTATCAGCCATAAGTCATCA | TGTTTGGTAAAAATAGCGGTTG |
| GB211976 | Imperfect | TGTTTTATC | 9 | 3 | 27 | TGAAAATCCAGAACTGTTCCA | TTAAGTATTTGCTCGGCTCCA |
| GB211977 | Imperfect | AATACA | 6 | 3 | 18 | TGTGCAAGAGATTAGCTGCCT | TGCTCTCTTGCCTAAGGTTCA |
| GB211983 | Imperfect | CTTAAT | 6 | 3 | 18 | GGGTATCAATCCTTGTGATTAAAAA | CACTGCATTTCAAGTCCATCC |
| GB211987 | Imperfect | TCAAAC | 6 | 3 | 18 | TCAACCGTTACATTAAACCATCA | CAATTTTTCCCCAAATTTTCAA |
| GB211995 | Imperfect | TAGTAC | 6 | 3 | 18 | CTCAAAGAGTTGCAATGGTGA | GCAAAGTCATGTGGTTTCTCC |
| GB211998 | Imperfect | ATTATA | 6 | 3 | 18 | TTAGCTTGGTTTTGGTCATGG | AGCTACATATTCCTAGCCATTTCA |
| GB212012 | Imperfect | AGTTGTC | 7 | 3 | 21 | CAGCAAGACAATTTGGAGGAA | TTGGCTATCAAGCGATTCAGT |
| GB212016 | Imperfect | AATACA | 6 | 3 | 18 | TGTCCCAAATTAAGTGAGAACAC | CCACATCAGGTTCTTTGTTGC |
| GB212017 | Imperfect | TTAAAA | 6 | 3 | 18 | CAAGTTCAAAATCAATGGTCAAA | CAAAGTGCAACAGCTGAATGA |
| GB212023 | Imperfect | AAATTG | 6 | 3 | 18 | TTTGATATTTGAATCAAGTTGTCCT | GACAAGGTGTATGATCGCAAAA |
| GB212025 | Imperfect | TATAAT | 6 | 3 | 18 | TGTAAGAATGTCTCCACGACT | CGTTTGATTCGCAATAAGGAA |
| GB212030 | Imperfect | ACAGAGC | 7 | 3 | 21 | GGGGAATGCTGTGACTACG | TGCAAACAATTCCATTTCCAT |
| GB212031 | Imperfect | AAAAGG | 6 | 3 | 18 | TTGACCAATGAACTATCCAGG | AAGGAGGGAAATAGAGCCGTT |
| GB212034 | Imperfect | AAGAAT | 6 | 3 | 18 | GACTCAATCCTAAATAAATGAGAAAA | CTTTTGCAGCTCAAGCAAGTT |
| GB212043 | Imperfect | AGAAAT | 6 | 3 | 18 | TTTGTGCTATTTAGGGGGAAGA | TCTCAATGATTAGGTTTGTTTATTCT |
| GB212050 | Imperfect | TTGGAA | 6 | 3 | 18 | TGTTGTGTTTGAAGAAATGAAGTG | GTCTTGAACAGCAAAGGTTGG |
| GB212055 | Imperfect | CAATTTA | 7 | 3 | 21 | TTGAACAAAGTCCATGTTTTATGG | CTGCTCCCTTTTTGCTCTTTT |
| GB212059 | Imperfect | AATATT | 6 | 3 | 18 | TTTTTGGTATTCGTTGCCG | GAAACACTTTCGACCCCACTA |
| GB212075 | Imperfect | TC | 2 | 11 | 22 | TGTTAGTGCTTGGAATATTTGTCA | AGTTTGGGACAGTTTTTGGGT |
| GB212079 | Imperfect | GGACTT | 6 | 3 | 18 | TAACGCCTAGGTCAAGGTTCA | CCAAGTCAGATTGTCCTCCAA |
| GB212080 | Imperfect | GGCGCGG | 7 | 3 | 21 | CACTCTAGAAAGTGCTGTGGC | CCAAGTCAGATTGTCCTCCAA |
| GB212094 | Imperfect | TTAGGAT | 7 | 3 | 21 | AAGCTTAGTCCCCCAAGAACA | ACAATCTCCATGCAACCAGTTA |
| GB212098 | Imperfect | AAACTA | 6 | 3 | 18 | TGGATAAATTAGAAGTTGGGATG | ATTCATGGAAACACAGTTCGC |
| GB212101 | Imperfect | TGAAATA | 7 | 3 | 21 | GCTAGCTTTGTCTTCATTTGTTT | CGATTTGAAACCTCGCTTATG |
| GB212104 | Imperfect | CCAAAT | 6 | 3 | 18 | ATCGGTCTCTTTCCCTTGAT | CCAAGAAGTGTGAAATTGGGA |
| GB212111 | Imperfect | ATAATT | 6 | 3 | 18 | CCTCCTTTCTCTTCTTCTTCTTC | TTTCCAACACCGATGATTTTC |
| GB212113 | Imperfect | ATTTAG | 6 | 3 | 18 | TGCTTTCTCTTCTTATTCTTTTT | AAATGCCCACCTATGAGCAG |
| GB212119 | Imperfect | CCTTCT | 6 | 3 | 18 | CCGACCTCAATTTCATCAACA | GGCACTGAAATGCAAGTTCTC |
| GB212123 | Imperfect | CTCCTT | 6 | 3 | 18 | CACTCTCTTCTGCTTCTTCATCC | TCCAATTTCACCGTTCATCTC |
| GB212124 | Imperfect | TACAAT | 6 | 3 | 18 | CGGATTCAAACGTCATCATCT | TCCAATTTCACCGTTCATCTC |
| GB212134 | Imperfect | TTCTAC | 6 | 3 | 18 | CGACTTCTGAACACGGTCGTA | TTGGTGTGTACCTAACCAGGC |
| GB212139 | Imperfect | TTTTAATC | 8 | 3 | 24 | ACATGTTGGGCTAAATGCGTA | GAGGAGTAGATGTTGGGGGAG |
| GB212140 | Imperfect | TAAAAA | 6 | 3 | 18 | TGAGAGACATTCTAATATTCATCATA | GAGGAGTAGATGTTGGGGGAG |
| GB212144 | Imperfect | ACCTTC | 6 | 3 | 18 | CAACCTCTTTCAATAAATTTCCCA | CTCAATGTTTGAAGGATCCGA |
| GB212146 | Imperfect | TTTAATT | 7 | 3 | 21 | GTCATATTTATATATTGTGCGTTT | CTATCGAACCATCCACCTGAA |
| GB212147 | Imperfect | CGATCA | 6 | 3 | 18 | CATGTTCTACGTCCCGATCAT | GGAATAGGGACAGGGACAGAG |
| GB212148 | Imperfect | CACGAT | 6 | 3 | 18 | TCTCTCGATCACGATCACGTT | AGGAATAGGGACAGGGACAGA |
| GB212150 | Imperfect | CTGTCC | 6 | 3 | 18 | GCCCGCGATCTCTTTCTCTA | TCGTGATCACAGAGAGGATAGAC |
| GB212162 | Imperfect | ATTAAT | 6 | 3 | 18 | AATGATTCCCATTAATTCATATAG | AAGGTTGGATTGAAAGTGGGT |
| GB212163 | Imperfect | AAGAAA | 6 | 3 | 18 | TCCTAAATTTTATCTTTTTGGAGA | AAGGTTGGATTGAAAGTGGGT |
| GB212166 | Imperfect | AACAACC | 7 | 3 | 21 | GTGGCGGACAACACTGTTACT | TAAGGAGGGACAAAAATGCAA |
| GB212168 | Imperfect | TAAAA | 5 | 4 | 20 | TAAGAGAGCGAAGAGGTGCTC | GCTCGGTGGTGAATCATAAAA |
| GB212172 | Imperfect | AGCCTT | 6 | 3 | 18 | AAGCTTCTTCTGTCTGCCATATC | CAGTTACCAGACACGCCTCAT |
| GB212189 | Imperfect | AATTTTC | 7 | 3 | 21 | TTGAAACATCCTCTTATCACTCAA | CGACACTGACACATGCGATTA |
| GB212190 | Imperfect | TATGTG | 6 | 3 | 18 | TTACTCGTTACTTCAAGCGTCG | TAAAGTGCGGTTCATGGTTTT |
| GB212192 | Imperfect | ATTCAAA | 7 | 3 | 21 | CTACTACCAGGTGGCAGAACC | ATTTATGAGCCATTGGAGCCT |
| GB212193 | Imperfect | CCAATT | 6 | 3 | 18 | ACCGCACATTCAAAATTCAAA | TTCTGCAATTTGACCATCTCC |
| GB212195 | Imperfect | AACATA | 6 | 3 | 18 | ACACCGGGGTACCTAATCAGT | AACAATAATGCCCCTTTTTCG |
| GB212206 | Imperfect | GGAGAA | 6 | 3 | 18 | GAAACGGTTGAACCAAGATGA | TGTCTTCATTTCTCCGAATGG |
| GB212209 | Imperfect | GAACT | 5 | 4 | 20 | TCAGAGGAATTGATTGAAGGG | ATGTGCCAAAGCATAATGGAC |
| GB212217 | Imperfect | ATTCAA | 6 | 3 | 18 | CTTCGATGGAAACAACGGTTA | AACACTCGTTTCTCGCTTTGA |
| GB212224 | Imperfect | ATT | 3 | 6 | 18 | GCGTACGTCGAGTACCCATAC | GCGCACAATTAGTGGAGAGAC |
| GB212232 | Imperfect | GATTTT | 6 | 3 | 18 | TTTGAATGAGAAGTTATTTTGTAG | CGATCGATTTTCAGGATTTGA |
| GB212238 | Imperfect | AATTAA | 6 | 3 | 18 | TCAATTCAGGTCGAGTAGATT | TTGCTTTGGAATCCGACATTA |
| GB212241 | Imperfect | CTAAAA | 6 | 3 | 18 | TTAGCAAATGAAGCCAACACC | TTCACCTTGGGTACGAGAAGA |
| GB212244 | Imperfect | TCTTCAAAG | 9 | 3 | 27 | GGATCAGTCTTTATTCATCTGGTC | CTGGTACATGATGTGATGCCA |
| GB212245 | Imperfect | AGCACATGT | 9 | 3 | 27 | CTGGCATCACATCATGTACCA | AGCTAAAAGGGGGAAAAA |
| GB212257 | Imperfect | TAAATT | 6 | 3 | 18 | ATGTGCTCCAATCTGCTCAAT | TGGGGATGAAATTAACGTCTG |
| GB212262 | Imperfect | AT | 2 | 10 | 20 | CACCGGTGTAATTTGATCTCTC | AAATCAAATCCAATCCAAGCC |
| GB212270 | Imperfect | AGGGGA | 6 | 3 | 18 | AATAGGGAAATGGAAGGCAAA | AAACACTTCAGTTCCTCCCGT |
| GB212271 | Imperfect | ATGTCT | 6 | 3 | 18 | TGACAAAAATGAATTGGACCTT | TTAGGTTGATAGTGGCCATGC |
| GB212272 | Imperfect | AAGGTG | 6 | 5 | 30 | CCAAGAGGTAGGAGACCAAGG | GTTGATAGTGGCCATGCATCT |
| GB212273 | Imperfect | ACACCA | 6 | 3 | 18 | CAACTACCAAACCCAAAATGC | GTGTTCATGCGGAAAAAGAAA |
| GB212275 | Imperfect | TTGGTT | 6 | 3 | 18 | ACGTGATTAAAGTGTCGAAGTGTT | GCCAAAATACATCAATGACGA |
| GB212277 | Imperfect | AATTCA | 6 | 3 | 18 | AACCCCGAGGGTGAAGAA | GAGTTTGGACCATTGTGCTGT |
| GB212280 | Imperfect | TGCTTG | 6 | 3 | 18 | TTTTCCTCTGTTTTCAGTGTATTTTC | TTTTGGTCGACATTTTGTGTG |
| GB212281 | Imperfect | AAAATA | 6 | 3 | 18 | AAAATGTTTGTCATAGATAACTCTC | ATATCGAAGACTTTGTGGGGC |
| GB212292 | Imperfect | ACTTCG | 6 | 3 | 18 | AAGCGTGGACTTAGCGAAATA | TGACCCATTTTTCGATCACAT |
| GB212293 | Imperfect | ATTCAA | 6 | 3 | 18 | CTTCGATGGATACAACGGCTA | TGACCCATTTTTCGATCACAT |
| GB212295 | Imperfect | AT | 2 | 25 | 50 | TCAGTTTTAAAGGCAATTCTCAA | ACCGTTAATCTTGATGGGTCC |
| GB212303 | Imperfect | AAGTTGA | 7 | 3 | 21 | TCCTTTCTGCAATAATATGTTCAA | AGCTTTGAAATCATGCAGTGG |
| GB212305 | Imperfect | TGCAAAT | 7 | 3 | 21 | TGGCTCAAGTTTGATGTGTTTT | AATGAGGGCACTGCTACCTTT |
| GB212309 | Imperfect | ACTTAA | 6 | 3 | 18 | GAAGTTTGCTTTAACACCGGTAA | TTCTCTACAATTTCTAGTTCTCTTTT |
| GB212316 | Imperfect | AAGGAA | 6 | 3 | 18 | TCTGATGTTGCAAAGAACCCT | GACACGTGGCAAAATGAAGAT |
| GB212321 | Imperfect | ATCATA | 6 | 3 | 18 | ATCTTCATAATGTGGTAAACGTACA | CAACTTTCAACATGTGCCTCA |
| GB212325 | Imperfect | GGAAAAA | 7 | 3 | 21 | AGAAATAGGGGAATCTGCACC | TCCCTTCTCATTTTGCAGAGA |
| GB212328 | Imperfect | AAT | 3 | 7 | 21 | ACATGCATGATTATATCTAGTAACTT | GGCGAAAATTCTTTTAGTGGG |
| GB212344 | Imperfect | TGATGG | 6 | 3 | 18 | ATCCTCATTGTTTTCGTGTGG | CTCCACCACCAATGTAGCATC |
| GB212350 | Imperfect | ATGAATA | 7 | 3 | 21 | CCATTTTAGTCAATCTTGCAATCA | AAGACTTAGGGGGAGTTTTCA |
| GB212351 | Imperfect | CAAAAG | 6 | 3 | 18 | TCGAACAAAAGGAAAAACTTGAA | TTCAGAAAAGCCTGGTGAAGA |
| GB212352 | Imperfect | AAGTAT | 6 | 3 | 18 | TCCAGCTATCTACAGCGAGAAG | TTCAGAAAAGCCTGGTGAAGA |
| GB212353 | Imperfect | CAAAAACG | 8 | 3 | 24 | AAAAACGAAGAGAACAAAGTCTTGA | TACCTACACCATTCGTCTCGC |
| GB212356 | Imperfect | AAAATTTG | 8 | 3 | 24 | TTTGATATTTGAGATTAGATGACA | ATTCAAGGGTCATCAAGGGAC |
| GB212357 | Imperfect | TGATATA | 7 | 3 | 21 | ACATGATGGTTGTTTTGTGCC | CCATCATATATAATTTTTCGCATTCA |
| GB212360 | Imperfect | TATTTAA | 7 | 3 | 21 | TGATTAGTCAATGTTAGATGATGTGA | AGGGAGCAATTCTTACTTGGG |
| GB212361 | Imperfect | AATTATA | 7 | 3 | 21 | GGAAGTGTGTGTGTGTGTGTG | GAACTGCCTTCCCAAAAAGAC |
| GB212362 | Imperfect | ATTTGATAT | 9 | 3 | 27 | CTGATTTTTGTGTTTGCTCTTCA | AAGAGCTCGGAAGTTTTCTGC |
| GB212366 | Imperfect | AATAGG | 6 | 3 | 18 | ACATGAATTACAAACAAGTATAATGC | CAATTTACCAAAATGCCCTCA |
| GB212371 | Imperfect | TTGTTCT | 7 | 3 | 21 | GGGTCTTGTTATCTTTCTTCTTTG | CATGGCTGGAAACAACAACTT |
| GB212373 | Imperfect | ATTCTT | 6 | 3 | 18 | CCTCTGATAGTATATCAAGTGTTCCT | TGGGTGCTGAGAGAGAGAGAG |
| GB212374 | Imperfect | CATAGC | 6 | 3 | 18 | TGCTTTTAGTTTTCGTGGCTG | CTTATCTGCACTGTTGGAGGC |
| GB212375 | Imperfect | TTATTAC | 7 | 3 | 21 | TCAACTCCTTGAGAGTCCCAT | TGCAGTTATGATGAGCAGCAC |
| GB212394 | Imperfect | CCAATT | 6 | 3 | 18 | TTTTGAAGAAATATGCCATGC | CCACCGGTATGAGTTGTGAGT |
| GB212396 | Imperfect | TTAGCA | 6 | 3 | 18 | GAAAGTTGTTGTTTGTGATTCTTTC | GTGGAAATTGGGAATGAGGAT |
| GB212419 | Imperfect | CACTCT | 6 | 3 | 18 | TCGTTTTTCTCACTCGATCAAA | AATTATGAACATACGTGCGCC |
| GB212422 | Imperfect | AGAAAAA | 7 | 3 | 21 | CCTACGTGTACATCTTGTCTTT | ATAATGAAGCCGACAAATGGA |
| GB212430 | Imperfect | ATCATG | 6 | 3 | 18 | TTCCATTAAATCAAAGGCAACA | CCATCTACCGGAGTTCACAAA |
| GB212439 | Imperfect | AAAAT | 5 | 4 | 20 | AGACAACCTCAACCGAACAAA | GGAAGCACTGAAAAATCTCCA |
| GB212442 | Imperfect | ACTTGTA | 7 | 3 | 21 | AATTACAGTGTGTGTTCTGAAAG | CAAAAGAAAATGCAACCCAAA |
| GB212447 | Imperfect | AATTAAA | 7 | 3 | 21 | AAGCTTAATTGCTTGTTCCAC | GGCCAAACGGTACCATAGTTT |
| GB212451 | Imperfect | ATCTTC | 6 | 3 | 18 | AACCTCGCATCATGTCTTGTC | ACGGAGATTTTGGGATCTTGT |
| GB212456 | Imperfect | TCCGTTGCGG | 10 | 3 | 30 | GATTTGAGATTCTCTGTTTCTTT | CGGGGTGAACATAAATCACAC |
| GB212459 | Imperfect | AAAGAT | 6 | 3 | 18 | GAGACAACGTTTTAGAGTGGCA | CTCCGCTCCCCTTTTATATTG |
| GB212463 | Imperfect | CTACAC | 6 | 3 | 18 | TGAGTGATATTGGCATCTCCC | CCTCAAGATGGGCTGTGTAAA |
| GB212467 | Imperfect | TTA | 3 | 7 | 21 | ATTTAACGTAAATTAGTCGATACA | ATGTGGCGGAATACATGGTTA |
| GB212468 | Imperfect | AGCACA | 6 | 3 | 18 | TCCAATTCATTGCTACAACACTT | GGCAATTGGCTTGTTAGGACT |
| GB212470 | Imperfect | ATAACAA | 7 | 3 | 21 | TTTTCTTTTCTAAAAATAACGAGG | AGGCAATTGGCTTGTTAGGAC |
| GB212475 | Imperfect | TTTGAA | 6 | 3 | 18 | TGGTTCTATATTTGAAGCATTGTTG | AACCTCTCATCCTCTGAGCAA |
| GB212481 | Imperfect | AATGTGC | 7 | 3 | 21 | ATAAGTGGGCATACCGGGTT | TGAAACTGCAACCGAAGGTAA |
| GB212485 | Imperfect | GTACCG | 6 | 3 | 18 | GGGTACTCCATTTTTGGGAGA | CCATAGGAGTACCATGCGTGT |
| GB212488 | Imperfect | GTACCG | 6 | 4 | 24 | AAAATACCGTACCCCGTACCA | TTGCCAATTCACCACTTTTTC |
| GB212489 | Imperfect | TA | 2 | 21 | 42 | AAATTCTACACCAAAAACTTGTATCA | GGGAAAGAAGGGCACAATATC |
| GB212492 | Imperfect | TTG | 3 | 15 | 45 | TGACACACTTTGAATCCCCAT | TCAAATGAGTGAATGCCATGA |
| GB212493 | Imperfect | ATTA | 4 | 6 | 24 | TGAACAATAAAGCTTTTGGCTAAG | TCAAATGAGTGAATGCCATGA |
| GB212495 | Imperfect | TAGGAT | 6 | 3 | 18 | GGGAAGAGATTCGGGATTTT | GGGAGAGAGCAACTCTCCATT |
| GB212496 | Imperfect | TATATG | 6 | 3 | 18 | TGCTTTCCGTATTTGGTATGTG | AGTCTCAGACGGGAAACCATC |
| GB212502 | Imperfect | GAACT | 5 | 4 | 20 | TCAGAGGAATTGATTGAAGGG | GGCGATGTGGCTCAGATAATA |
| GB212507 | Imperfect | TATTTT | 6 | 3 | 18 | TTAGCGTTAAGCAAATCCGTC | ATGGAGAGTTCGAGTGAGGGT |
| GB212510 | Imperfect | AAATAAAA | 8 | 3 | 24 | TGGAGATAAGAAATCTGTGTTGA | CTTAGTGAGTTCAGGGCAACG |
| GB212519 | Imperfect | CAAATT | 6 | 3 | 18 | GCCAAAACCTTTCACACAAAA | TGGTTTTACTTTTGGGGTGTG |
| GB212522 | Imperfect | TTGCAA | 6 | 3 | 18 | AAAATAATGAGGTGCATGGATT | AGAAAAATGGGGTTCGAGAAA |
| GB212523 | Imperfect | TTATAGC | 7 | 3 | 21 | GGGCCTAAGACTCTGTTTGGT | GTGTTTGGTAAAATTAGCGGTTG |
| GB212526 | Imperfect | TTTGGT | 6 | 3 | 18 | GGGAGAATTATTTGTTCCTTT | TAAGAACATGCATGCGTTGAC |
| GB212528 | Imperfect | TAAGAA | 6 | 3 | 18 | AACAAGAGACTTCTCAAACAAACT | CTCTGCAACGGTCATCATCTT |
| GB212529 | Imperfect | ATTAAAG | 7 | 3 | 21 | GCATTGAAAAATGTCCCTTGA | TTTCCCAGTGGCTTTCCTAAT |
| GB212530 | Imperfect | ATT | 3 | 6 | 18 | AGCTGAGCTAGCAGAGCGAAG | GGTGGATATGTCTGCGACTGT |
| GB212532 | Imperfect | CGACTT | 6 | 3 | 18 | ACCCTAATTCATTCATTCAATCT | GCCCTTAGTAATGCCGAACTT |
| GB212537 | Imperfect | TGATT | 5 | 4 | 20 | CCATTGATATGGTGTTTGTGTG | ACCCGAGAAACTCCAAGAAAA |
| GB212546 | Imperfect | AGTGGT | 6 | 3 | 18 | TTCTCGTTTAAAAACAACCAT | AATTGCATCCTTTCAATAAGTTCA |
| GB212547 | Imperfect | AGATAT | 6 | 3 | 18 | CAACTGTTTAAAATGGAAAATAC | ACGACATGTTCTATGGCTTGC |
| GB212557 | Imperfect | TTGGCC | 6 | 3 | 18 | CGTCCTTCATTTTAAATAATTC | ATCATGGCGATTGATTGTGAT |
| GB212558 | Imperfect | TTAGAA | 6 | 3 | 18 | AAAAAGGAATTTTGTCAGAAGAC | TCAATTGGTATCAGGAGCTGG |
| GB212564 | Imperfect | CAGATT | 6 | 3 | 18 | ATTTTCGGAGTTTGCTGCTCT | GCAACGGTAGCCATATTCAAA |
| GB212567 | Imperfect | GGACTT | 6 | 3 | 18 | GATGCCTATGTCGAGGTTCAA | AACAATTCTCCACCTTGCCTT |
| GB212569 | Imperfect | GGCGCCA | 7 | 3 | 21 | CGGAAATTTTGAAGAACATCTG | AAGACCCTAGCCTTGTGTGGT |
| GB212571 | Imperfect | TGAGTT | 6 | 3 | 18 | TGAGTCATATTCAACACTGAAA | ATGTGCAGGCACTTTAGGGTA |
| GB212576 | Imperfect | ATCCCC | 6 | 4 | 24 | ATTTTTGCCTCCATCCCC | TTTTTGCAACGTCTTTTTGGT |
| GB212577 | Imperfect | ATTGGTA | 7 | 3 | 21 | TGCATAATCATACAAAACCAAA | GATTCCCCGAATCCGTCTAT |
| GB212581 | Imperfect | TC | 2 | 19 | 38 | AGCGGACTTCCACTTTCTAATG | GCACAAGCAAATTGGTTTCAT |
| GB212589 | Imperfect | GGAAAGAAA | 9 | 3 | 27 | ATGCTCTCTGGTTGTGGATTG | CGCATCAACAGTTCCATTGT |
| GB212594 | Imperfect | AACTTA | 6 | 3 | 18 | GTGTTGCATGAGTGAGTTTGC | AATCGAAAACCTAGCCAAAGC |
| GB212609 | Imperfect | TCTAAT | 6 | 3 | 18 | CGTTGAAAAACAATTTGACACA | ATGGGGCAGTGTTAGTGAATG |
| GB212615 | Imperfect | AATTGAA | 7 | 3 | 21 | AAAAGAAAAAGCTAGTTTCCATGT | TGTTTGGTATCACGTTGGGTT |
| GB212618 | Imperfect | TGAAGA | 6 | 3 | 18 | ACGCTAGAACCGGTGAGGTT | CGCTTAATAAAATCGAACGCA |
| GB212623 | Imperfect | GAGATTT | 7 | 3 | 21 | CCAACGTGAAATGTAAACAGC | AGGCTGATCCCAATGAAAAGT |
| GB212632 | Imperfect | AACAACC | 7 | 3 | 21 | TGGTGGACAACACTGTTACTGA | AGAAAGGAGGGACAAAAATGC |
| GB212636 | Imperfect | CAT | 3 | 11 | 33 | AAGCCTTTAAATGTTGTTTTACCT | AGTGTTTGTGGGTTGTTCAGG |
| GB212637 | Imperfect | AAGAAA | 6 | 3 | 18 | TTGCAAAGTTGGATAGAATTAAGAAA | AGTGTTTGTGGGTTGTTCAGG |
| GB212642 | Imperfect | TATTGT | 6 | 3 | 18 | TGTTTCGTGTTCCTAATCTACGTT | AGCATGGGTGAGTGTCATAGG |
| GB212650 | Imperfect | TAAAAA | 6 | 3 | 18 | AGAATCAATGAACATCGTGTGA | TTATGCCCCATCCTCTCTTCT |
| GB212659 | Imperfect | AACCAA | 6 | 3 | 18 | CAAAACCAAAACTAGATGAACACG | CCCTCTCATTCACGAAATTCA |
| GB212662 | Imperfect | TCGTTG | 6 | 3 | 18 | TCACGAACCTAAAGCAAGAGA | GTCCCTCACCGAAATCTACCT |
| GB212666 | Imperfect | ATTATG | 6 | 3 | 18 | GCCATATCAATGTAATCTTTGGGT | TCGGAGCCTTCCAAAATAACT |
| GB212669 | Imperfect | TCACCA | 6 | 3 | 18 | TCTTCGTTTCTTCATTTTCGG | TCTTCCCTTTTTCGTTCGATT |
| GB212670 | Imperfect | GCGAGCG | 7 | 4 | 28 | TCGAAAAGAACAAGTGAAGAAGA | CCAAAAATGGCCCAAATAGAT |
| GB212674 | Imperfect | ATTGAA | 6 | 3 | 18 | GGAGAAGAGATTATGATGAAGGC | CAGAAAACATCATCTTCCCCA |
| GB212681 | Imperfect | TTAATA | 6 | 3 | 18 | ATCTTGGACCAATTGGGTTTT | CACACCTCTTGTCACCAAACA |
| GB212683 | Imperfect | CATATT | 6 | 3 | 18 | GGGGCACCTAATCAGTCATAA | CTCACCTCAGAGTGTCCTTGC |
| GB212685 | Imperfect | TGGAGC | 6 | 3 | 18 | TGTAATTTGGAATTGGGGATG | GCTGACTCAGAAACCAAATGC |
| GB212686 | Imperfect | ATTGAA | 6 | 3 | 18 | ATGGAGCTGGAGCTGGTAAAT | ACGACACGATATTGCAAGAGG |
| GB212690 | Imperfect | TCAGAAAA | 8 | 3 | 24 | GCAAGGTGGAGATTTGTGAAG | AGCCAGAAGCCACAGCTATTT |
| GB212693 | Imperfect | TTAGAGT | 7 | 3 | 21 | CCTTCTCCTTTCTTTTTGTTGG | CAAGGAAAACAAGGCCACTC |
| GB212700 | Imperfect | AAATAATAAT | 10 | 3 | 30 | TGGCATACAATTGATACAACAAA | CAAAGCAAGCCTGACAAAATC |
| GB212714 | Imperfect | TA | 2 | 9 | 18 | GTGTGTTCGACAACAGCAATG | GCGAACCCTTCAAACGTTACT |
| GB212719 | Imperfect | AAAGAG | 6 | 3 | 18 | GGACTATAGCTGATTTTCTGGT | TGGAGAATCAAGGAGGGAACT |
| GB212720 | Imperfect | TATGC | 5 | 4 | 20 | TGTTCCTCGATTTTCTTAATGTCA | TTTCATAACGAGGTTGCTGCT |
| GB212731 | Imperfect | TGTTTTG | 7 | 3 | 21 | CTCAGCTTCATCCAAGTTGTTC | GCCAAATAAACCGAAGAAAGG |
| GB212734 | Imperfect | AACGTTT | 7 | 3 | 21 | CGACGAACGTAAACCACAAAA | AGGTTTCGGATGGGAGTTTTA |
| GB212739 | Imperfect | ACTTGA | 6 | 3 | 18 | AGACCCAAAGTTCGAGGTTGT | AGGAATGCTTAACAAGTGCCC |
| GB212742 | Imperfect | GCCAT | 5 | 4 | 20 | CGTCATATGTAACTAGGGTTGGG | TTAAACAGGCTACAAGGCCAG |
| GB212746 | Imperfect | TTTGTG | 6 | 3 | 18 | GACATTATCAGATTTTGCATTTGAG | TCACAACCAAAGAGGAAGTTCA |
| GB212755 | Imperfect | ATAACA | 6 | 3 | 18 | AAAAGACGAGAAGAGAGCACG | TGTAACCCTAATCCTAGTGGTGAA |
| GB212768 | Imperfect | GAGTTT | 6 | 3 | 18 | CCAATGATGATGTTTTCGGAG | TAGAACCACGTTGCAGAAAGG |
| GB212779 | Imperfect | TAAGTTA | 7 | 3 | 21 | CATTCTTTTGTGTGAGAAATTGG | TCATGAATCAACATAAAACAAACC |
| GB212784 | Imperfect | TATTTT | 6 | 3 | 18 | TGCATATAAAAATTCCTACACAAAGG | ATCCGAGTTTCTGGCTATGGT |
| GB212789 | Imperfect | ACGTCT | 6 | 3 | 18 | CTATTGATGGGGAACAAAGAGG | GCTACAAGCTCGGCATAAACA |
| GB212794 | Imperfect | ATGTTT | 6 | 3 | 18 | TTAGAATCCCAATTCCAACTCC | AAATGGGCCTTAGCCTTAACA |
| GB212812 | Imperfect | GAATTA | 6 | 3 | 18 | TTTAGCTGAAAATCAACTTGAAGA | AGATCAGCCATTAGGGGAAGA |
| GB212815 | Imperfect | TTATATA | 7 | 3 | 21 | TCAGTCAATCCAAATATTGTTCGT | CGATTATCCGGTAAAAAGAACC |
| GB212821 | Imperfect | AAAATCA | 7 | 3 | 21 | GCAATGTCCCAAACTTCTGAG | CCACAATCATTGACGGGTAAG |
| GB212826 | Imperfect | ACATATAT | 8 | 3 | 24 | TCGTTCTGCACAGTACGTTAGAC | GAATTTGGTTGCGCAAGTATC |
| GB212836 | Imperfect | ATCTCC | 6 | 3 | 18 | CTCTCTTCCACACCTCCTCCT | GGTCTTAAGCTCGATTCCGAT |
| GB212859 | Imperfect | TTTCTT | 6 | 3 | 18 | GTTGATGCAACATTGGCACTT | CTGGAAAGAAACATTACACCTGG |
| GB212863 | Imperfect | TATTTT | 6 | 3 | 18 | AGTGACACTTATTTTGGAATAGGA | ATTTTGAAAAATTGGGTTCGG |
| GB212868 | Imperfect | TTTCAT | 6 | 3 | 18 | TTGCTTGACAAAATCACACCA | AATGCTGCTGGCTTATACGAA |
| GB212888 | Imperfect | TGGAAT | 6 | 3 | 18 | CAAAATGGATGAGTGATAAATTGAA | ATGACCGTTGCAGAGAGAGAG |
| GB212889 | Imperfect | ATTCTT | 6 | 3 | 18 | CCTCTGATAGTATATCAAGTGTTCCT | TAACGAGAGGGTGCTGAGAGA |
| GB212892 | Imperfect | ACCA | 4 | 7 | 28 | CAACATCATGGTTTTTGATGTCTT | TATTTTTGGTCAGGGAAAGGG |
| GB212896 | Imperfect | AAGAAA | 6 | 3 | 18 | AGATGGGCACATGAAAACAAG | AGCCTCCGTAACTCATTTGGT |
| GB212908 | Imperfect | TC | 2 | 9 | 18 | ATGCGCATGTTCCGTA | GCGCTTACCATTACACCAAAA |
| GB212923 | Imperfect | AATTTT | 6 | 3 | 18 | AATCAATTTTAACCGTCTGATTT | CGGTTGTAACTGCTTCACACA |
| GB212925 | Imperfect | TTAATGTT | 8 | 3 | 24 | CTCATGTTCTTGGTCTTGCTT | TCACAAGTTCTCCCCAAACTG |
| GB212929 | Imperfect | AT | 2 | 17 | 34 | AGATTGATTATGGAGAAGAGATGA | GGAGTTGCTAACGTACACCCA |
| GB212937 | Imperfect | TAATTG | 6 | 3 | 18 | TGTCACTAGACAGAAGGAAAGGAG | TCTGCATATTTTGGTGCTGGT |
| GB212942 | Imperfect | TTGCTC | 6 | 3 | 18 | ATTTGCAACAGCTTCATGGAC | TGGTAAAGCAGAATGCCAAAG |
| GB212948 | Imperfect | ATAAAA | 6 | 3 | 18 | AGCTTGCAAGTGCGCTAAAC | TTTGGTGTCTCCGATCAAAAG |
| GB212958 | Imperfect | TATTTTAC | 8 | 3 | 24 | ACATTCTTCTACTATTGTATTCATGT | CTTAGCTTTCCAACGCAACTG |
| GB212966 | Imperfect | CCAAAT | 6 | 3 | 18 | CCTTGATCAATCAATGTTAGACG | TAGCCGATGCTTTGAGTAGGA |
| GB212967 | Imperfect | ATGTTT | 6 | 3 | 18 | TTAGAATCCCAATTCCAACTCC | ATGGGCCTTAGCCTTAACAGA |
| GB212979 | Imperfect | TTTTAT | 6 | 3 | 18 | TGATCAAACATAGCTTATTTTCAAAG | AAATCCCTCTCGTTCAGGAAA |
| GB212988 | Imperfect | TTTAAT | 6 | 3 | 18 | CTATAGTGTATTATAATTTTTCCTGG | CAAATAGTTTCATAACAAAATTCGGA |
| GB212991 | Imperfect | ATACAA | 6 | 3 | 18 | GCCCAAATAAATATTTCTCCA | TCTGGCCTCCATGACTTTTTA |
| GB212993 | Imperfect | TGATTT | 6 | 3 | 18 | GATTTTGAATTATGTTGGACAAA | AATCCAAACAGGCCCTTAATTT |
| GB212994 | Imperfect | TATTAAT | 7 | 3 | 21 | TCATCCTGCACAAAGGAATGT | CCTTGTGAATGGCCAATATGT |
| GB212996 | Imperfect | ACAATG | 6 | 3 | 18 | CCAAAAGGTTTGGTGATTTTC | CCACATACCAGTTCCGAACAC |
| GB212999 | Imperfect | TTTCTC | 6 | 3 | 18 | AAAATCGTGTTTGGAAAGGCT | CACCAGCTAGCAACTTTGGAG |
| GB213005 | Imperfect | TGTACA | 6 | 3 | 18 | TGAACAAATCAAAATATACATCTCCA | TTCCAATTTTTATCGGTGTCG |
| GB213007 | Imperfect | AGAATGA | 7 | 3 | 21 | ATGGGTAAGAAGAGGCCGAT | GGAACTGTTGTAGTCTTGTAGATG |
| GB213012 | Imperfect | CACAAT | 6 | 3 | 18 | CAACCCTGTTATCTTTCCAGTT | TCTTTCGATGCATTCAGGAAC |
| GB213013 | Imperfect | TTGCAT | 6 | 3 | 18 | CACGTGTCATTTTCTCGTGAGT | ATGAGTGCAAAGAAAAGTCGG |
| GB213017 | Imperfect | GCCATG | 6 | 3 | 18 | CCAACAAAAGACAAGAACGACA | CTTTAAACAGAAACCGTCCGA |
| GB213026 | Imperfect | TTATTT | 6 | 3 | 18 | CCTTTATTTTTCAATTGCACCC | AGGTTAAGCTAACCGGTGTCC |
| GB213031 | Imperfect | AAT | 3 | 6 | 18 | TGATAAGAAAATATGTAGCCCAC | GGTTTTGCAGTCTCAGCCTAA |
| GB213035 | Imperfect | CTAGTATACA | 10 | 3 | 30 | TCTCCAAGATTATGACAGTTCG | TCCATAATTCATCCCAAACCA |
| GB213036 | Imperfect | TTGATT | 6 | 3 | 18 | TGGAGGTGTATTTGAGTGGAAA | CCCACAGAAATTATCCGGTTT |
| GB213051 | Imperfect | TTAATAC | 7 | 3 | 21 | TCTGTTGGGACATTTTACTTTTTG | ACTTTGGGACGGAGTACAGGT |
| GB213056 | Imperfect | TAGAACC | 7 | 3 | 21 | TGAGAAGCAAAGCATCAGAGG | TCTCCAAAACGATTCGTCCTA |
| GB213062 | Imperfect | AAATTG | 6 | 3 | 18 | TTTGATATTTGAATCAAGTTGTCCT | CATACCTTGAGTGAACCGCAT |
| GB213063 | Imperfect | TCAATT | 6 | 3 | 18 | AGCCTACATTCCAACAACCG | CCCGGATGTCCCTAAAGAATA |
| GB213064 | Imperfect | AAGAGA | 6 | 3 | 18 | TGTTTTAGACGATGTGTGGGA | CAGTTCATACGGAGGCATTGT |
| GB213082 | Imperfect | TAATTA | 6 | 3 | 18 | CACAAATCTCAGATGAATCTTATT | TTATTGATGAGGTGAGGACCG |
| GB213085 | Imperfect | AATATC | 6 | 3 | 18 | GCCATCAGGCTGTGCATATAA | TTTCGATGATGCGGTTAAATC |
| GB213095 | Imperfect | TCATAG | 6 | 3 | 18 | GGGAACTTTGTCAGAAGACCT | TCAATTGGTATCAGGAGCTGG |
| GB213096 | Imperfect | TGT | 3 | 11 | 33 | AGAATTGGAATCATAGTCATAGTTCA | TCAATTGGTATCAGGAGCTGG |
| GB213119 | Imperfect | TAAAATA | 7 | 3 | 21 | CAAAGAAAATTAAGCTGAACAAATCA | ATGCCATGATTCACCAGCTAC |
| GB213199 | Imperfect | AAATCT | 6 | 3 | 18 | TGTGTTGGTTGCGTAAATGC | GGGTCACTTTGATTGGAACATT |
| GB213202 | Imperfect | AAATCT | 6 | 3 | 18 | TGTGTTGGTTGCGTAAATGC | TGTCACATGCACATTTTTCAG |
| GB213356 | Imperfect | ATACATA | 7 | 3 | 21 | CTGCAATCAGGTAAATAATCGTT | ATCGCACAGTGTAGCCAGAGT |
| GB213407 | Imperfect | TGTTTT | 6 | 3 | 18 | TCACTTATTCACAGTCGACAAAAA | AAATACAGCAGTGCCCACAAC |
| GB213408 | Imperfect | AAAGAA | 6 | 3 | 18 | CTCCAATCCTTTAAACTGCCA | TCACATCACATCAAGCCAGAA |
| GB213410 | Imperfect | TGCAATT | 7 | 3 | 21 | CAACTGCAACATTGAGGTCAC | TGAATGGAACTTGCAGGAACT |
| GB213412 | Imperfect | TGTTTT | 6 | 3 | 18 | CGGTCACTAGGTTACTTGACAAAA | TCAAATAAACCGGGCATGATA |
| GB213413 | Imperfect | AAAGAA | 6 | 3 | 18 | TCTCCAATCCTTTAAAATGTCA | GAATCCACCAAGAGTGTTCCA |
| GB213414 | Imperfect | TGCAATT | 7 | 3 | 21 | TTGTTGCAACTGCAAAATTGA | TCAATTTGGACCACACCTTGT |
| GB213445 | Imperfect | ATGAGG | 6 | 3 | 18 | TTCGTTGACACTATCTTGTTTGA | GCTTGCTTTTGACAACCACAT |
| GB213447 | Imperfect | CACCGG | 6 | 3 | 18 | CTAATTACTCTGCCTGAGCCG | AACATAGCCAATGATGAGACCC |
| NF206357 | Perfect | GAA | 3 | 6 | 18 | AAGGGCTTCTTTTAGAGGAGGA | TACCATCATCATCTTCACCACC |
| NF206374 | Perfect | ATC | 3 | 6 | 18 | TTTTGCCAAACCAAAAACATC | AAGGTTCCTTGAGGCCTTTCT |
| NF206375 | Perfect | TGA | 3 | 7 | 21 | GAGAGGGTACTTTCAGATGATGG | CCTACTCCTAGCCCAACCATA |
| NF206383 | Perfect | AGTTTG | 6 | 3 | 18 | AAGGGATGACGCTACG | TAAAGACCAACAAGACCCTGC |
| NF206403 | Perfect | AC | 2 | 9 | 18 | CAGACCACAAAACAATCAATG | CCAAACGATGTTGTGTGCTAA |
| NF206405 | Perfect | TGT | 3 | 7 | 21 | TCAACAAAATTCCCTAACCCA | CATCATTTCAACCACCTCCAT |
| NF206410 | Perfect | ATGATGA | 7 | 3 | 21 | TTTTTGAAACTGAAAACAAGGAC | GGTCCCACGTACATATTCAGA |
| NF206425 | Perfect | TCATCATCT | 9 | 2 | 18 | GTCCCAATCGTCATCATCATC | ATCGGCAAACAAAGATGAGTCT |
| NF206428 | Perfect | AGATGA | 6 | 3 | 18 | TCGGCAAACAAAGATGAGTCT | ACCAACACTGCATGGTCTCTC |
| NF206429 | Perfect | GATGAAGAT | 9 | 2 | 18 | ATGAGTCTGGGCAGCTGAAT | GATGCTCTGTGAGGCAGCTAT |
| NF206488 | Perfect | CA | 2 | 9 | 18 | GAAGTAGATTGTATCATTGTTCCAGC | GTTATTTTGGCACATCCCTCC |
| NF206523 | Perfect | GAT | 3 | 8 | 24 | CTTACTGGGTTCGTGTTCGTT | TGAGTCCTGAGTAACTATTTTAGAAC |
| NF206543 | Perfect | TTG | 3 | 6 | 18 | CAAATGAAGCATGAAGTGGGT | ACCTTGCCTTTCCACTTTCTC |
| NF206548 | Perfect | CA | 2 | 11 | 22 | CCAAGATTCTGATTTTGGCAAC | GACCAATGAAAGACCCATCAA |
| NF206565 | Perfect | TCA | 3 | 6 | 18 | CATCAAGGCATATTGTCAATGAA | GTCCTGAGTAACCGCACACTG |
| NF206595 | Perfect | TATGTGTGTG | 10 | 2 | 20 | CCTGAGTAATGCTACTACATGGC | GAAAGGGCCTAATTTGGATCA |
| NF206601 | Perfect | TTC | 3 | 12 | 36 | TCGATATGACACTCCCAGCTT | AGACCAAAAAGCCAAGGAAAA |
| NF206618 | Perfect | GAAATTGAA | 9 | 2 | 18 | AATATGCGCCTATCTTACAGCG | GCCCTTGATGAGTCCTGAGTA |
| NF206663 | Perfect | CAT | 3 | 7 | 21 | TACAACAACAACGGGAAAACC | CCAAAACCTGTTGTTGAAGATG |
| NF206678 | Perfect | TGATGATGG | 9 | 2 | 18 | CATTGCCGTAAAATGCAAAAA | ATACAGGTTTTCGTGCCTCAA |
| NF206692 | Perfect | ATC | 3 | 9 | 27 | AAATCACATTTATTGCAACCGT | ATGTGTTGCTTCCATTCTTGC |
| NF206700 | Perfect | TCA | 3 | 6 | 18 | TGACCATACTTGACCTCATTCC | AGTAACAGCAACTGGCGATGT |
| NF206701 | Perfect | TGA | 3 | 6 | 18 | GCATATTTGGCACGTTGTAATG | TGAATCAAATTTGGCAAGAGC |
| NF206716 | Perfect | TGA | 3 | 6 | 18 | GGAATTGATTGATGTTTTGTTT | ATCGGAAGCTATCGAATCACA |
| NF206728 | Perfect | GA | 2 | 9 | 18 | TGAGAGAGAGGAGATATTTTGGAGA | ATGAGTCCTGAGTAAACCCCC |
| NF206730 | Perfect | TGA | 3 | 7 | 21 | CTGTTTGTGTTTTGGTGATGG | GTCCTGAGTAAACCCCCTAGC |
| NF206737 | Perfect | ATG | 3 | 6 | 18 | GAGTCAATGCTGGAGTCAGAAA | AACCATCTCCCATAGACCTCA |
| NF206746 | Perfect | AACTATAAG | 9 | 2 | 18 | GTGCTCAGCCCAATAGGGGTTT | CCTCGACAATTTTCCCC |
| NF206748 | Perfect | CAT | 3 | 9 | 27 | ACTTCTCAATTTCTCCGCTCC | CCAAGCAAGTACATCATCACTAAATC |
| NF206770 | Perfect | CTCATCATC | 9 | 2 | 18 | GAATAATGCCTTGATTCTGCAT | CCCCCTCCTCCTGTTCTTACT |
| NF206851 | Perfect | ATC | 3 | 6 | 18 | TTGTTGTCAATCTTCTTTGTTTAGAA | GAAGCTGGTGATGGTAGGGTT |
| NF206924 | Perfect | GAT | 3 | 6 | 18 | GAATCAGAATCTAATGAATATGATGA | ACCCACACCAAACCCTGATA |
| NF206938 | Perfect | AGTCAGTCA | 9 | 2 | 18 | GAGTCAAGACTACCAATCCGTACA | GGAATAAAGCATATTCCCCCA |
| NF206941 | Perfect | TGCATC | 6 | 3 | 18 | ACCCTCTTTCCATAAGCGGTA | GGCCAAAACTATTTGGAGTCA |
| NF206954 | Perfect | GAT | 3 | 9 | 27 | TCAGAGAGGGTACTTTCAGATG | GATTTGTCAAAACCTATATCTATGA |
| NF206997 | Perfect | GAT | 3 | 6 | 18 | CCTCAGAGGATGAGGATTCTG | CTACCCCCTCCTAGCACTACAA |
| NF207000 | Perfect | GAT | 3 | 6 | 18 | TTAGCAGGATCGATTCGTAAAT | GTCCTGAGTAAACGAAAAGGG |
| NF207005 | Perfect | CAT | 3 | 8 | 24 | CCTGCCTAACCAAAAACACAA | TGTTCTCAGCAACAGCTTCCT |
| NF207038 | Perfect | ATC | 3 | 6 | 18 | TCCTCTTGGAAAACCTCTTGG | GTTGAACGATGATGATGGTGA |
| NF207085 | Perfect | ATAACTAAC | 9 | 2 | 18 | GCAATCCAAGTTAGTTACAACA | TTCTGTTATTAGCTTATGCATTC |
| NF207092 | Perfect | TG | 2 | 10 | 20 | AGTAATTATTAGGAGGGGGAGCC | GTGTGCAGCTGGACTGTATGA |
| NF207193 | Perfect | TCACAAT | 7 | 3 | 21 | CCTCAATCTCCAACTCCCAC | CCAATGGTTGGTTTGTTTCAA |
| NF207217 | Perfect | TCAGTA | 6 | 3 | 18 | CTTTCATTACTCGTGCTACTATTAT | GCCCTTGATGAGTCCTGAGTA |
| NF207239 | Perfect | CA | 2 | 9 | 18 | TGAGTAACACACTCAATTGCAAAG | TTTTTGATTTTTGCCCCAATA |
| NF207240 | Perfect | ATAACTAAC | 9 | 2 | 18 | GTTAGTTACAACCAACTCTATCCA | TCTGAGACTGATCTTGAAAACACA |
| NF207247 | Perfect | AGTG | 4 | 14 | 56 | TGATGTGTTTGACTGACATGG | TAATCACAGCCACCCATCTGT |
| NF207254 | Perfect | CA | 2 | 12 | 24 | CGTCAAAAACAGAGAACAGCC | ATTCCAAGTGTTTATTTGCCTG |
| NF207265 | Perfect | TTGCTTTCTG | 10 | 2 | 20 | TTTGCATTACCAAAGTATCTTTCTC | TTGTGCAATGAACACAAAAGC |
| NF207315 | Perfect | CAATTAT | 7 | 3 | 21 | CCATAAGTTCATAATGGAATGCAA | GAGTAATAGGGATGAGTGGCCT |
| NF207316 | Perfect | ACACC | 5 | 10 | 50 | GAGGTTAGTCAATTAGGACTTCTCT | TCATTAGGGGACCATTGCTTT |
| NF207319 | Perfect | AG | 2 | 13 | 26 | TATATATACACACACACACACACAC | GATGAGTCCTGAGTAATGAAGAAC |
| NF207366 | Perfect | AAG | 3 | 6 | 18 | TGTGTGTGTACAACCGGAATC | ACCATTGATTGAGGAGCATGA |
| NF207376 | Perfect | CCTTCCCTT | 9 | 2 | 18 | CCACCCTATGTCTCACCTTCC | CCTAAAATGTTTGCGCCCTAT |
| NF207391 | Perfect | AAC | 3 | 6 | 18 | TCTTCAAATACCAACAACCAACA | AACGACTCCAACCTTTTGTCA |
| NF207404 | Perfect | TCAC | 4 | 5 | 20 | CATTCCACCGCTTCTTTCTC | GCATGCGTTGGTTAGAGAGTG |
| NF207410 | Perfect | AG | 2 | 9 | 18 | GTTACTTGGTTTGATCACGCA | ACACAGACTACACGCTCGTCTT |
| NF207415 | Perfect | TGTGT | 5 | 5 | 25 | AAAATCTCTACCATGACTTTGCTTTA | CCAGAACAATAGAAAGATCATCA |
| NF207443 | Perfect | CA | 2 | 9 | 18 | TCAAAGGATTGTTCCAATCAAA | TTAGGGTCAGTGGATTGCAGA |
| NF207451 | Imperfect | ATGTTG | 6 | 3 | 18 | AATGCTGATGATGCTGCTGAT | AACAGAGACAGGGCAACACAT |
| NF207454 | Imperfect | ATGTTG | 6 | 3 | 18 | CTGATGATGCTGCTGATGGT | AAAAAGAGACAGGGCAACACA |
| NF207471 | Imperfect | GAAGAT | 6 | 3 | 18 | AGAACTCTGAGCACGAAGCTG | GGACCTTACACTCACCTGCCT |
| NF207475 | Imperfect | AAGATG | 6 | 3 | 18 | TGAGCACGAAGATGACTCAGA | CACTCACCTGCCTTTTTGATT |
| NF207476 | Imperfect | TCATCT | 6 | 3 | 18 | TCAGAGCGAGAAAGATTAGCAA | AAGTGCAACAAACCAGGACAC |
| NF207481 | Imperfect | GCTGAA | 6 | 3 | 18 | AGGAAGCTGAGGAAGAAGGGT | CCAAACAGGGCAACATAACAT |
| NF207502 | Imperfect | TGA | 3 | 6 | 18 | GAACGATGATTTCAGTGACGG | ATCATCATCACCAGAGCCTTG |
| NF207515 | Imperfect | GGGGAT | 6 | 3 | 18 | GAAGGCGAGGAAGCTGATAAT | ACAGCTGACAGGAGAACATGAA |
| NF207530 | Imperfect | TATGATGA | 8 | 3 | 24 | TGATTGACATTATGCTTGCTTG | ATAGGCCGCTCAACCCTATTA |
| NF207539 | Imperfect | GCTGAA | 6 | 3 | 18 | AAGAGGAAGCTGAGGAAGCAG | CAAACAGGGCAACAAATTCAT |
| NF207541 | Imperfect | GCTGAA | 6 | 3 | 18 | AAGCTGAGGAAGCAGGGTTAG | TAAAAGGAGCCACTGGAATCA |
| NF207542 | Imperfect | CAACAC | 6 | 3 | 18 | TCTTCAACATCATCCCCAGC | ATTTTTGCCCTGAAGCAAGAG |
| NF207543 | Imperfect | CTTCAG | 6 | 3 | 18 | ATCAGCAGCTTCAACACCAAC | TGAGAAGGTCATTTTTGCCC |
| NF207545 | Imperfect | AGACTC | 6 | 3 | 18 | AAGAAGAGCTTTCTGGCAACA | TGAACCTGCTTCAGAGTCTTCTC |
| NF207546 | Imperfect | CAT | 3 | 7 | 21 | AAACCCATTAGTGTAGTTCTGCCT | GTAACTCAGTAGAGTCAATGCTGGA |
| NF207547 | Imperfect | GAT | 3 | 7 | 21 | TCAATGCTGGAGTCAGAAAGAG | ACCCTCTAAAGAACCATCTCCC |
| NF207552 | Imperfect | CCCAATTGA | 9 | 3 | 27 | ATTGAATGGATTGAGGCCTTT | TGTGGATGATGATGATGAAGG |
| NF207555 | Imperfect | GAT | 3 | 8 | 24 | ACAAGTGATGGTTTGGCAGAT | CTGAGTAAACCAGCCCTGAAA |
| NF207564 | Imperfect | TGAAGA | 6 | 3 | 18 | CGAGGAAAATGTTGCTGAAGA | AATATTTACCGGCGCTTCACT |
| NF207576 | Imperfect | GAT | 3 | 8 | 24 | TTTTTGAAACTGAAAACAAGGA | TGTAGGTCCCACGTACATATTCA |
| NF207581 | Imperfect | GT | 2 | 9 | 18 | CCGTGAGATTGGAAAATTGAA | CATGTTCGTCAAACCCAAATC |
| NF207582 | Imperfect | GGGATTT | 7 | 3 | 21 | GCGCGAAGGAAAATGAAAT | CAGACACTTCATCCCTTCCAA |
| NF207585 | Imperfect | AACTCAG | 7 | 3 | 21 | CGGAATCATAATCATTGGTGG | CCTGAGTAAGCTCATGAACCA |
| NF207586 | Imperfect | GAA | 3 | 6 | 18 | CTTTGATGTGTTGATGAGGGG | CGAAAAACGTGCCAAAGATAA |
| NF207587 | Imperfect | TTTCCA | 6 | 3 | 18 | TTCTGTTTATCTTTGGCACGTTT | AACCTCATCCGTAACATGGGT |
| NF207589 | Imperfect | TCTCCA | 6 | 3 | 18 | ACCTACCAACACTGCATGGTC | GGCAAACAAAGATGAGTCTGG |
| NF207590 | Imperfect | ATC | 3 | 13 | 39 | CTGTTACGGTTTTCATCGTCC | AAACAAAGATGAGTCTGGGCA |
| NF207591 | Imperfect | GAT | 3 | 10 | 30 | TCAGCAGATGAAGATGAAGATGA | TCTGTGAGGCAGCTATTCCAC |
| NF207596 | Imperfect | ATCTCA | 6 | 3 | 18 | TTTCAATGGATTTTGAATTGATCT | TGGTTTTGGTTTTGCTCTATC |
| NF207598 | Imperfect | ATGAAG | 6 | 3 | 18 | ATGCAAATGATGTTCCAATGC | GAGATCCCCTTATCCCTAGCA |
| NF207599 | Imperfect | TCTTCA | 6 | 3 | 18 | TATTCATCATCAGAGCCTTCCG | TAATCCAGCTTGATGACAGT |
| NF207608 | Imperfect | TG | 2 | 9 | 18 | GGTGACTCTATAGTTGTACTGGGCT | CAGCTATTGCTTGGAGCCTTA |
| NF207609 | Imperfect | GTTTTTT | 7 | 3 | 21 | CAACGTACTGTTGCTGTTGTT | GAATTCGCCCTTGATGAGTC |
| NF207610 | Imperfect | TTTCAT | 6 | 3 | 18 | GGCCCAGTAGTGGATGAATAGA | GCCCTTGATGAGTCCTGAGTA |
| NF207611 | Imperfect | TCAT | 4 | 5 | 20 | TTTTTCTCTCAATTTCATAACTGTG | GCCCTTGATGAGTCCTGAGTAA |
| NF207612 | Imperfect | AATGAA | 6 | 3 | 18 | TGATCACAGTTATGAATTTGAGAGA | GCCCTTGATGAGTCCTGAGTA |
| NF207616 | Imperfect | TGTGT | 5 | 7 | 35 | CCATGACTTTGCTTTATCACTTGT | TTGTGTCATGAAAACCAGAACA |
| NF207617 | Imperfect | TGTGTT | 6 | 3 | 18 | CGCTGTTGTGCATTGAGTCTA | TACGTCTGGGGGATACCAATC |
| NF207626 | Imperfect | CAGACA | 6 | 3 | 18 | TGCTTATATATCACAGACACACACA | TATTGTTTTCTTAGAACATTGATACC |
| NF207628 | Imperfect | TGA | 3 | 6 | 18 | TCACTAAGTAATAGGAAAGGGTTTTG | TTGGAGGCAAATCATGCTAAC |
| NF207629 | Imperfect | GACTTT | 6 | 3 | 18 | TTCGCTTGAGCTACTTTCAACA | AAAGGGAAAGCACAATGACAA |
| NF207632 | Imperfect | CACCA | 5 | 6 | 30 | TTCTCTACATCATATTTATGGCAATG | CACAAGTGTCATTAGGGGACC |
| NF207637 | Imperfect | ATG | 3 | 6 | 18 | GGGTTGATGCTCAAACACATA | TCTCATCCTGAGCAACACCAT |
| NF207640 | Imperfect | TTG | 3 | 7 | 21 | GTGAGTGATTTAGATCGGTTTTGA | AATCCAATCACACACCCAATG |
| NF207645 | Imperfect | ACCACCC | 7 | 3 | 21 | ATTTTCATTGCCGATCATCAC | TCACATAATGATATTGCATCACAA |
| NF207648 | Imperfect | CAT | 3 | 7 | 21 | GAACCTGATCTTTCCTCACCC | AGGAATAAATGTGTTGAGAGGCA |
| NF207649 | Imperfect | TGATGC | 6 | 3 | 18 | GAGGGGAAGCTGAGTTTA | TCACCCAAGACCATTCATTTT |
| NF207651 | Imperfect | TCATTA | 6 | 3 | 18 | CTGCTGACCTACTTGCTGAAA | GGCAAGTTATAATGTTGGATCG |
| NF207654 | Imperfect | GT | 2 | 9 | 18 | TGAGTCCTGAGTAATACTATATGATG | AACCAATAGCAAAGCAATAAGCA |
| NF207657 | Imperfect | TTGTTT | 6 | 3 | 18 | TCTCTTCTCTCGTCTGTTTTGTTCT | AACACACACACCACAAACACC |
| NF207658 | Imperfect | TGTTTC | 6 | 3 | 18 | TTGTTTTTGTTTTTCTGTTGC | AGTAAACGCAACAACATCAAGC |
| NF207667 | Imperfect | TTACAAC | 7 | 3 | 21 | AGTCTGATGGCTACTGATTGC | TACTGGCTTTGGACTGGTAGGT |
| NF207668 | Imperfect | CAT | 3 | 9 | 27 | CTCCTGGTGACTCTGACGTTG | TTCCTTTGTCTCCACTGCAAG |
| NF207671 | Imperfect | TGAGAAA | 7 | 3 | 21 | CGTTACGATGACGACGATGAG | TCCTAACATTTGTTGAATCTAACGG |
| NF207683 | Imperfect | TCTTGG | 6 | 3 | 18 | CTGAGGTGGTGTTTGGCTG | AACACCTCTCCGTCACCAATA |
| NF207686 | Imperfect | AC | 2 | 19 | 38 | ATTAACACCAATGTCATCCAA | TGGGACGGAACTAGTATCAAAT |
| NF207687 | Imperfect | TG | 2 | 12 | 24 | CAAATTGTCTTGTTATCACATTCACT | GAATTCGCCCTTGATGAGTC |
| NF207692 | Imperfect | TGTGTT | 6 | 3 | 18 | GCCGTTATTGTGCTTGAGTCT | GAGATCCCATCTCACTCCCTT |
| NF207700 | Imperfect | ATC | 3 | 10 | 30 | TGAGTCCTGAGTAAACTAAAGAAAAA | CCGTCGTTTCCTAACAAGGTA |
| NF207721 | Imperfect | TGGTGT | 6 | 3 | 18 | GGGTTAGTGGTTGAAGCTGAA | AACAAACAAACAGGGCAACAA |
| NF207722 | Imperfect | GAT | 3 | 6 | 18 | AGGGTTTGAAAGAAGAAATTGTG | ATCACTCTCCTGTCCCTCCAT |
| NF207726 | Imperfect | TG | 2 | 10 | 20 | TGGTGAATTATGGGAATCACG | GTCCTGAGTAATTAGCAGGTCTA |
| NF207731 | Imperfect | TG | 2 | 12 | 24 | CCACACAGGCATACACATTCA | AGCCTTTGAGCTCCATTTTCA |
| NF207744 | Imperfect | TTCGGTTT | 8 | 3 | 24 | CTCTAAAAATTGTTGGGCCAC | TGTGCTTTTACACCAAACACG |
| NF207745 | Imperfect | TCA | 3 | 6 | 18 | CTTCATAGCATTTCTTAGCATTGAA | GCCCTTGATGAGTCCTGAGTA |
| NF207748 | Imperfect | TCA | 3 | 7 | 21 | TTTTCATCAAGGCATATTGTCA | GAGTCCTGAGTAACCGCACAC |
| NF207759 | Imperfect | TC | 2 | 9 | 18 | GAGATAACACAACCACACACACA | AACATAACAACGTGGGTGGAA |
| NF207767 | Imperfect | TTC | 3 | 6 | 18 | CAGAACACAACTTTTACCCAAACA | AGAGAGTGAGTGAGTCACGGG |
| NF207783 | Imperfect | AACACAG | 7 | 3 | 21 | ATCAGCACGCCTCAGGTATTAT | AGGCCTAGTGCTGTGTCCATA |
| NF207789 | Imperfect | GTGTAG | 6 | 3 | 18 | AGAAATACCTCAATCTGTGAGGAAAA | GAGTAACACCAGAGACCGGAG |
| NF207796 | Imperfect | AG | 2 | 14 | 28 | AATGAGCGAAAAACACAAATG | CTAGGCTCAGGAGACGTCAAT |
| NF207802 | Imperfect | GATGAG | 6 | 3 | 18 | AGCATAGTTGCTCATTTGTTTTT | TTTCCACCATGTCAGTGCTTT |
| NF207808 | Imperfect | TCA | 3 | 7 | 21 | CGACAAGAAACGATTGATGAAG | ACATTTGGATCAACCATGGAA |
| NF207816 | Imperfect | GAT | 3 | 6 | 18 | TTCAATGATGGTGGGTATATTGA | TCCTGAGTAATATAAACATATGAACA |
| NF207829 | Imperfect | ATC | 3 | 6 | 18 | GAGACGAATTGTGCTCCAAGA | TTCTCCAAAACCATTCATCCA |
| NF207839 | Imperfect | AAAGAATA | 8 | 3 | 24 | TTGTGTTCCACACTATCCAAACA | GAGTCCTGAGTAAAGGGCCAC |
| NF207846 | Imperfect | TATGTG | 6 | 3 | 18 | ACAAGGTAAAGCCATCTTTGGT | TTTCTTCTCTCCCCTTTCGAG |
| NF207857 | Imperfect | TCAGAAC | 7 | 3 | 21 | CGGAATCATAATCATTGGTGG | ATTCGCCCTTGATGAGTCC |
| NF207883 | Imperfect | GATGAG | 6 | 3 | 18 | CCAAACAGGTGGTGAATCTGA | CGCAGGTTTGAAACATACCAT |
| NF207887 | Imperfect | CAT | 3 | 6 | 18 | CAGGGATCTGATTTTCATTGTG | TCACGCAGTCAGTCCAACC |
| NF207891 | Imperfect | GAT | 3 | 6 | 18 | ACATTGCCGTAAAATGCAAAA | ATACAGGTTTTCGTGCCTCAA |
| NF207895 | Imperfect | GATGCT | 6 | 3 | 18 | CCTGAGTAAATCAGAAAATGCAGA | CAATTCCTTGAGCCTCCTCTT |
| NF207905 | Imperfect | GAT | 3 | 9 | 27 | TTCACTGCTAGAGTCAGAGAGGG | AAATAAAAATCGGTGGCGACT |
| NF207906 | Imperfect | AGAACC | 6 | 3 | 18 | GCCGAAAAATACGGAAAAAGA | CAATAACAGAAACACAAAATGACA |
| NF207907 | Imperfect | TCACCGTCG | 9 | 3 | 27 | CATCATCATCATCATCATCGTC | TTGGAAAACGATTTCTGAAGC |
| NF207912 | Imperfect | TATTT | 5 | 4 | 20 | TGTCCCTCTTTATACCATCTTT | TTTGAGATTGCTCTGGTAAAT |
| NF207913 | Imperfect | TCTTCA | 6 | 3 | 18 | ATCATGGTGGAATTGAAGTGG | CTGCGCGAGAATTTTTGTACT |
| NF207918 | Imperfect | GAT | 3 | 7 | 21 | TCAATGCTGGAGTCAGAAAGAG | TGCTCAAACCTCTACCTCTCA |
| NF207924 | Imperfect | AAGCTG | 6 | 3 | 18 | CGGCAAATTTACATGGAGAAC | CCCCAATTCCATTCTCTAACAG |
| NF207926 | Imperfect | GAT | 3 | 9 | 27 | TTTTGAAACTGAAAACGAGGAGT | TCAGATTTGATTTTTCTACTTATACC |
| NF207929 | Imperfect | TTA | 3 | 6 | 18 | TAACCCAGAGCCTTCTCAAGC | TTGTAATGTGGTTTGTCACCG |
| NF207930 | Imperfect | CAAAACA | 7 | 3 | 21 | ATTTGTTCTTTCCCAGCAACA | CTGCCCCTAAGATTCAAAAGG |
| NF207938 | Imperfect | GTGATT | 6 | 3 | 18 | GTGACTTAATTGATAGTTGATAATTG | GATGAGTCCTGAGTAACGAGA |
| NF207942 | Imperfect | GGTTTT | 6 | 3 | 18 | GTCAACCCAATCCGGAATACT | GAAAAGGAGGAATTAGGGTTTCA |
| NF207943 | Imperfect | GGATTT | 6 | 3 | 18 | CGCAAAAATACTCCCTCTGATT | TGAAAATTAGTTTCCGCATCA |
| NF207944 | Imperfect | TGA | 3 | 6 | 18 | GAGAGATTGGGTTCATGTGGT | TGAGTCCTGAGTAAACCCCCT |
| NF207945 | Imperfect | TGGTGA | 6 | 3 | 18 | TGTGGTTATTGATGATGATGATG | TGAGTCCTGAGTAAACCCCCTA |
| NF207954 | Imperfect | ATGA | 4 | 5 | 20 | GTTTGGTTAGATCGATGGACG | AAAGGCACACAATCCTCATTCT |
| NF207956 | Imperfect | TGA | 3 | 9 | 27 | AGTAAACGCACAAGTCACGGT | GCTGCTTCATTCATTTCAACC |
| NF207957 | Imperfect | TGATAA | 6 | 4 | 24 | GATGATGATGATGATGATAGATGTTG | GCTGCTTCATTCATTTCAACC |
| NF207958 | Imperfect | TTCAGT | 6 | 3 | 18 | TGATAATGATAATACATTGTTGGA | ATGGCCATTCTAGCCACAGAT |
| NF207965 | Imperfect | TGAATC | 6 | 3 | 18 | AGAGGGCCAATCGTACTCAAA | GAATTCGCCCTTGATGAGTC |
| NF207967 | Imperfect | CAT | 3 | 10 | 30 | ACTTCTCAATTTCTCCGCTCC | TCCAAGCAAGTACATCATCACTA |
| NF207969 | Imperfect | TGGAAG | 6 | 3 | 18 | TGGAGGCTTCTTGAACAAAAT | TCCCAATTCCCCTAATATTGC |
| NF207971 | Imperfect | TGCAGG | 6 | 3 | 18 | GCCATGAAGCAGGAGGAA | AACAAACAGGGCAACAAATCA |
| NF207972 | Imperfect | GAT | 3 | 6 | 18 | CCATCATCAAATCAGTTGCTTC | TCTTTAGGAGCTTCCGCCTTA |
| NF207974 | Imperfect | TTCTCA | 6 | 3 | 18 | CTCTCAAGCTCCTCCACCAC | TGAACTGAATGAGGTTTCGGA |
| NF207986 | Imperfect | TATTT | 5 | 4 | 20 | TTGGTATGGTTTCATGTCCCT | GCTCTGGTAAATAGATTGGATCGT |
| NF207987 | Imperfect | GTG | 3 | 7 | 21 | GGGGTTTGTTTGAAGGTGTTC | ACAATGAGAACGACAACGTCC |
| NF207988 | Imperfect | TCTTGG | 6 | 3 | 18 | TGGTGGACTGAGGTGGTGT | ACAATGAGAACGACAACGTCC |
| NF207990 | Imperfect | ATG | 3 | 8 | 24 | GATGAGTCCTGAGTAATATTTGTA | TCTCCTCTTTCACCTCCACCT |
| NF207992 | Imperfect | GGAGGT | 6 | 3 | 18 | TTCATGGTTGTTTTGTTTGATTC | TCCAACATCATTACGTTCCAA |
| NF207995 | Imperfect | TCATGA | 6 | 3 | 18 | ACATCACCATCAACTCCATCA | TGGAGGAAAACACTGAGACTGA |
| NF207997 | Imperfect | ATGATTG | 7 | 3 | 21 | AGGTCGAGATCTCTGTGCTGA | TTCGTGTGTTTTGTTTTCTGTGT |
| NF208027 | Imperfect | ACCAAG | 6 | 3 | 18 | GTCTACGTACCCCACCCTTTC | GGGGTTTGTTTGAAGGTGTTC |
| NF208036 | Imperfect | ATAATCA | 7 | 3 | 21 | AACTCGTTCAAGTTGGTGCTC | ATGAGTCCTGAGTAAAGAAGAAAA |
| NF208055 | Imperfect | CAT | 3 | 6 | 18 | AGGAGCACAACTGTAGTAACAC | GTTAATATCCAGCTTCGGCCT |
| NF208060 | Imperfect | TGA | 3 | 7 | 21 | TCACTAAGTAATAGGAAAGGGTTTTG | CTCTTCGGTGATTGAAAAACG |
| NF208062 | Imperfect | GAT | 3 | 12 | 36 | GAATCTGATTATAGTGCTGACCCTG | AAAATGATTGATAACACTAACCTTCA |
| NF208064 | Imperfect | GAT | 3 | 16 | 48 | CAGAGAGGGTACCTTCAGA | TTATAATGGGAATTTTTCCAGCC |
| NF208065 | Imperfect | CAT | 3 | 7 | 21 | TGGATCCAGACACTTCCATCT | AGTAATGATGCTGGTGATGGTG |
| NF208067 | Imperfect | CGAGGA | 6 | 3 | 18 | TGCAAAACATCTTAAAGGAGACAG | TGCTGACAACGGAATATCATC |
| NF208096 | Imperfect | CTTC | 4 | 6 | 24 | TGAAGATGATTCAGGATGTGAAG | AGGCCCATTTATATAGCCAGC |
| NF208097 | Imperfect | GAGAAG | 6 | 3 | 18 | ATGATGATGGTGGGTGAAGAA | TGAGTCCTGAGTAAAGGAACAAA |
| NF208116 | Imperfect | AGGTGC | 6 | 3 | 18 | CAACCTCAGGTGGAGTCACA | ACCACCATCATCATCACCATC |
| NF208138 | Imperfect | TCTTCA | 6 | 3 | 18 | ATCATGGTGGAATTGAAGTGG | GGTTCGGATTTTGAAGATTGG |
| NF208140 | Imperfect | AGTCAGTCA | 9 | 3 | 27 | CCAATCCGTACAACTATGAATGG | TTCCCCCAAATGATTAGTACG |
| NF208148 | Imperfect | ATGTTG | 6 | 3 | 18 | TGAATGCTGATGATGCTGCT | AAAAGGACCCACTGGAATCAG |
| NF208150 | Imperfect | TTCTGC | 6 | 3 | 18 | TGTGTACAACATTTTGCTGCTG | GATTGGATTCTGTGATGCTGAC |
| NF208159 | Imperfect | TGA | 3 | 8 | 24 | AGAGGGTACTTTCAGATGAAGATG | GCCCAACCATAGATTTGTCA |
| NF208163 | Imperfect | TCACAA | 6 | 3 | 18 | AGATTTTCAAATATGCAGGAAACAC | TCAACCTTGTTGATTCCATTT |
| NF208164 | Imperfect | ACAAGG | 6 | 3 | 18 | TGGGATATCTTCAATGGTCCC | CTTTCTTCTGCCCTTCTGTCC |
| NF208171 | Imperfect | ATG | 3 | 6 | 18 | TCAGAAAGGGTACTTTCAGATG | CCAACCATAGATTTATCAAAACC |
| NF208187 | Imperfect | GATGAA | 6 | 3 | 18 | AGATGCCCTTGAAGTGGAAGA | TCCTGAGTAAGGTATCTAAGAAATCG |
| NF208201 | Imperfect | GTGTGA | 6 | 3 | 18 | ACGAGGCGAACGGAGTT | TCATCATCATCAGAAGAAGCAA |
| NF208206 | Imperfect | TGGTGA | 6 | 3 | 18 | TGATGATGATGCTTCTACTGC | CATTGATTCACCAGATTCCCA |
| NF208210 | Imperfect | AACACAG | 7 | 3 | 21 | GCTCTGGATACCACTTGTTGG | AGGCCTAGTGCTGTGTCCAT |
| NF208218 | Imperfect | GATTCA | 6 | 3 | 18 | CAACCACTTCTGATCAAGAGAAC | CCTGAGTAATTCAGAGCGAGAA |
| NF208224 | Imperfect | TTC | 3 | 6 | 18 | AGATTTTGTTGCTTCATCAGC | AAGAAAGGTGGCAAGACTGGT |
| NF208227 | Imperfect | GAGGGT | 6 | 3 | 18 | GTGGACTCTTGGGGATGGTA | AGCATCCCACTTTCTTTCTCC |
| NF208229 | Imperfect | CAT | 3 | 9 | 27 | CTGCCTAACCAAAAACACAAAA | GGTGAATTCAGGTTGGAGTGA |
| NF208240 | Imperfect | TCTTCA | 6 | 3 | 18 | GCCAATGATCATGGTAGAATTG | GTCTGCGCGAGAATTTTTGTA |
| NF208249 | Imperfect | AAGATG | 6 | 3 | 18 | GGGGATGATAAGTTGAGGGATA | TCCTCACCGAACATAACCAAC |
| NF208263 | Imperfect | ACATGA | 6 | 3 | 18 | TCCAACAATCCATATGCTTCC | AAGATCGGTTGAATCATGCTG |
| NF208264 | Imperfect | CAT | 3 | 6 | 18 | GAACTCAAAAGCAAAACCTTCA | CTGAGTAAAACAATCGCGTCC |
| NF208265 | Imperfect | TGAACA | 6 | 4 | 24 | TCATCATCATCAAGAACAAACTCA | CCTGAGTAAAACAATCGCGTC |
| NF208271 | Imperfect | GAAGCT | 6 | 3 | 18 | CAGAACCAAAAGGGGTGGAG | GACATTCGAACTTTCACCAACT |
| NF208275 | Imperfect | ATGTTG | 6 | 3 | 18 | CTGATGATGCTGCTGATGGT | TAAAAGGACCCACTGGAATCA |
| NF208276 | Imperfect | GCTGAA | 6 | 3 | 18 | CCCTGAAGCAAGAGGAAGC | CCAGCTGCATCACCATCA |
| NF208278 | Imperfect | AAG | 3 | 6 | 18 | TGCACATCATCAATCACTAATTCT | TTATTGACACCCTCCTTTCCC |
| NF208286 | Imperfect | TGA | 3 | 6 | 18 | GAAGATGGCACACTTTGTGGT | CACATCAACCGTTCGTCTCAT |
| NF208312 | Imperfect | AGTA | 4 | 5 | 20 | GCACTCACACAACACATCACAC | GCCCTTGATGAGTCCTGAGTAA |
| NF208318 | Imperfect | TTTGTTTT | 8 | 3 | 24 | CCAAAACAACCGGATCAGAAT | CACACACACAAACACCAAAAA |
| NF208336 | Imperfect | AGAAAT | 6 | 3 | 18 | AATCTGGGTCCAAGGAGAAAC | GATGTCCTAACGTGTTCGACG |
| NF208355 | Imperfect | ATGGACC | 7 | 3 | 21 | ACAATTGCGGAGATCAATCAG | GTCACACACACAAACACAAA |
| NF208359 | Imperfect | TC | 2 | 11 | 22 | TCTCCGCCACTCTACTCTTCA | CCAAACAAAACCTAAGCTTTCAA |
| NF208375 | Imperfect | CCA | 3 | 6 | 18 | TCACTCTCATTCCCTTCACAT | CCACTATCGAAAACGGTGGT |
| NF208376 | Imperfect | ACGGCG | 6 | 3 | 18 | AGTGAGAACAGCGTGTGGTG | AAATTGCTCTCACTCAACCCA |
| NF208382 | Imperfect | AC | 2 | 9 | 18 | ACACAGACCACAAAACAATCAA | CCAAACGATGTTGTGTGCTAA |
| NF208429 | Imperfect | TGTGTTG | 7 | 3 | 21 | CGGAAAAGGGTTTGTTTTGTT | AAAACCCCTCCTTTTTCTCAAC |
| NF208430 | Imperfect | GTGAGAA | 7 | 3 | 21 | TGTTGTTTGTGTTGTGTTTGAT | TAATATTCCACCAAAACCCCTC |
| NF208459 | Imperfect | AG | 2 | 9 | 18 | TGCAATCATGGAGAAAACAGA | ACAAACAAAGCCACACCACAT |
| NF208486 | Imperfect | TCAGTA | 6 | 4 | 24 | TGCTACTATTATTACCAGTATCTACG | ATTCGCCCTTGATGAGTCCT |
| NF208496 | Imperfect | AAGAAA | 6 | 3 | 18 | GGTGTCTGGTGGTGAGAGAGA | TTCAAATCAATTTTTGTTCCC |
| NF208504 | Imperfect | AGGAGA | 6 | 3 | 18 | AATGGAGATGGTGGAGACGG | CAATGGCTAGAACCAAAAACC |
| NF208509 | Imperfect | AGAAGAA | 7 | 3 | 21 | TCTCGTTTATCTCAACAAGGG | ACAAACAAAGGAGGCATTTCA |
| NF208513 | Imperfect | TTACAA | 6 | 3 | 18 | TGGGTTTAGGTGTTAATACGGG | TGTACCCCACACACACACACT |
| NF208523 | Imperfect | GGTGTGGATG | 10 | 3 | 30 | CAAGTGGAGGAGGAAAGAAGG | CACCAACACCAAACAAAGACC |
| NF208526 | Imperfect | AGTG | 4 | 15 | 60 | ATGTGTTTGACTGACATGGT | TGAGTAATCACAGCCACCCAT |
| NF208543 | Imperfect | GGTTTT | 6 | 4 | 24 | TCTCACATTACTAAAAACCACAATGA | TCCTGAGTAAACCCAAAACGA |
| NF208546 | Imperfect | CAA | 3 | 6 | 18 | CCAAACAACAACAAAAGAAAACA | ACGAAAAGCATGAAACCACAA |
| NF208555 | Imperfect | TTGCTTTCTG | 10 | 3 | 30 | TGCATTACCAAAGTATCTTTCTCTCT | TTGTGCAATGAACACAAAAGC |
| NF208558 | Imperfect | TTGTAA | 6 | 3 | 18 | AACGGGTAACATATTTTTCTTATG | TCTCTCATATCAATGGCCCAG |
| NF208561 | Imperfect | TTTC | 4 | 5 | 20 | ACACCTCTTTCTTTTGTTTCCC | TCTCTCATATCAATGGCCCAG |
| NF208566 | Imperfect | TG | 2 | 9 | 18 | GCAATTCCCTGCATATCTGAAC | TTTGGCTTCAAAGAGCCTAGC |
| NF208567 | Imperfect | GTGTGTAT | 8 | 3 | 24 | TTGAAGCCAAAACAAAAGGG | AAGCACACCTCATGAGAAGACA |
| NF208570 | Imperfect | ATAATG | 6 | 3 | 18 | AGGTGATTACTTTTCCACTTGA | GTCCATAAGCTTCATTTGCCA |
| NF208579 | Imperfect | CTTGCGGTAT | 10 | 3 | 30 | ATGTGCTTAGGTGTGTGTGTGTT | TAGCATGATCACTGCAAGCG |
| NF208587 | Imperfect | GAGGAA | 6 | 3 | 18 | GCGATAATAAAAAGAAGAATGAGGA | TTGCACCGTGAGAAACAGAAT |
| NF208592 | Imperfect | AGAGAA | 6 | 3 | 18 | ATGAGAGAAAGAGAAGTTAAGAGAAA | GTCCTGAGTAATCTACCATGGCTT |
| NF208593 | Imperfect | CCTTTC | 6 | 3 | 18 | CCAAATACTCATTCCCTTCAACA | TCACCCAATTCAACAACAACA |
| NF208602 | Imperfect | CA | 2 | 11 | 22 | ATCCTCTTTCACACTCAACATCA | AATGGATGTGCAATGCAATTA |
| NF208611 | Imperfect | ACACC | 5 | 11 | 55 | GGTTAGTCAATTAGGACTTCTCT | ACAAGTGTCATTAGGGGACCA |
| NF208616 | Imperfect | AGAAAA | 6 | 3 | 18 | GACGACCAACACAACGTCAC | AACCAAAAATCACCCCTCTAAA |
| NF208619 | Imperfect | GGTGAA | 6 | 3 | 18 | GGAAGCCATATTTTGGTACTGG | TGGAATTGCATCAGACAACAC |
| NF208625 | Imperfect | GTTTGT | 6 | 4 | 24 | CAATGTGTTTGTGTGTTTGTGTG | CCAATCGGTGAATTCCAAAAA |
| NF208628 | Imperfect | TGGTT | 5 | 4 | 20 | TCTTTAGATTATTGAGACAATGTTTG | AATCCACAGGTTCCAAATCAA |
| NF208632 | Imperfect | GTTTGA | 6 | 3 | 18 | GAAGGTGTTGAGGTGATGTGG | TACCGTCAACACGGAGTGTCT |
| NF208638 | Imperfect | TTATGA | 6 | 3 | 18 | CATGAATCATAATGAATGAGTTTGG | CACACAACACAGCACTCTTGC |
| NF208640 | Imperfect | AGT | 3 | 7 | 21 | AAAGGTGGTGTGTGTGTTTGA | GACGAAGAATGCTTCAACAACTT |
| NF208653 | Imperfect | ATCAC | 5 | 4 | 20 | ACAGCTCCCCCTTAACACCT | ATGATCTGCACTGCTCTGGAT |
| NF208657 | Imperfect | ACTC | 4 | 5 | 20 | TTTGCATTTTGCTAAATCGTTG | TCCTGAGTAAAGAGGCGTGAG |
| NF208671 | Imperfect | TGG | 3 | 6 | 18 | TTGATGAGTCCTGAGTAAAACCT | CTTCACCACCATCACCTAAGC |
| NF208685 | Imperfect | AGACACC | 7 | 3 | 21 | CCAACATCTATCAAGCACCAA | TTACTTTGATTGGCAAACGGT |
| NF208695 | Imperfect | GA | 2 | 11 | 22 | ACAAAGAAATGTGAAGAACAGAAGAG | GAGTCCTGAGTAACCGCATAGC |
| NF208696 | Imperfect | ACCAACA | 7 | 3 | 21 | CAAAGATCCGAGTTCTTCAAA | CGGTTTAGTTTTCCAACATCACT |
| NF208698 | Imperfect | ACACCCAC | 8 | 3 | 24 | CAAGGAAGGAAAGAAGGAAGG | TCCTGAGTAACACAACCGCAT |
| NF208708 | Imperfect | ACCAACA | 7 | 3 | 21 | CAAAGATCCGAGTTCTTCAAA | AACGACTCCAACCTTTTGTCAT |
| NF208712 | Imperfect | AACAAA | 6 | 3 | 18 | CTTCACTTCACTCATCTTATTCATCA | TCAAGTTTTCAATGATGGTGGA |
| NF208714 | Imperfect | TTGTGG | 6 | 3 | 18 | TGAGTGAGTGTGTTCTGGTGG | TGAAGATTCTGAACGCAAACC |
| NF208722 | Imperfect | TTTTTA | 6 | 3 | 18 | TGCAAGAATTTTTGAATGATTGAT | CCTGAGTAATCAAAACCGCC |
| NF208743 | Imperfect | TTATTT | 6 | 3 | 18 | GCCCTTGATGAGTCCTGAGTA | CTGAGTAACAACATGGCAGCA |
| NF208745 | Imperfect | TGTTCTA | 7 | 3 | 21 | TGGTGTGTGCTGTTGTTTATTAG | TGAAAACCACACTAATGGATGC |
| NF208751 | Imperfect | GTTTGG | 6 | 3 | 18 | CCGTATCTTTTGGAATTTGAT | TCTCGTGTTGGCAAAGACAC |
